# Brain metabolite levels in remitted psychotic depression with consideration of effects of antipsychotic medication

**DOI:** 10.1007/s11682-023-00807-0

**Published:** 2023-11-02

**Authors:** Hideaki Tani, Iska Moxon-Emre, Natalie J. Forde, Nicholas H. Neufeld, Kathleen S. Bingham, Ellen M. Whyte, Barnett S. Meyers, George S. Alexopoulos, Matthew J. Hoptman, Anthony J. Rothschild, Hiroyuki Uchida, Alastair J. Flint, Benoit H. Mulsant, Aristotle N. Voineskos

**Affiliations:** 1 Centre for Addiction and Mental Health and Department of Psychiatry, Temerty Faculty of Medicine, University of Toronto, Toronto, Canada; 2 Department of Neuropsychiatry, Keio University School of Medicine, Tokyo, Japan; 3 Department of Cognitive Neuroscience, Donders Institute for Brain, Cognition and Behaviour, Radboud University Nijmegen Medical Centre, Nijmegen, Netherlands; 4 University Health Network and Department of Psychiatry, Temerty Faculty of Medicine, University of Toronto, Toronto, Canada; 5 Department of Psychiatry, University of Pittsburgh School of Medicine and UPMC Western Psychiatric Hospital, Pittsburgh, PA USA; 6 Department of Psychiatry, Weill Medical College of Cornell University and New York Presbyterian Hospital, White Plains, NY USA; 7 Nathan Kline Institute for Psychiatric Research, Orangeburg, NY USA; 8 Department of Psychiatry, New York University School of Medicine, New York, NY USA; 9 University of Massachusetts Medical School and UMass Memorial Health Care, Worcester, MA USA

**Keywords:** Antipsychotics, Brain metabolites, Magnetic resonance spectroscopy, Placebo, Randomized controlled trial

## Abstract

**Background:**

The neurobiology of psychotic depression is not well understood and can be confounded by antipsychotics. Magnetic resonance spectroscopy (MRS) is an ideal tool to measure brain metabolites non-invasively. We cross-sectionally assessed brain metabolites in patients with remitted psychotic depression and controls. We also longitudinally assessed the effects of olanzapine versus placebo on brain metabolites.

**Methods:**

Following remission, patients with psychotic depression were randomized to continue sertraline + olanzapine (*n* = 15) or switched to sertraline + placebo (*n* = 18), at which point they completed an MRS scan. Patients completed a second scan either 36 weeks later, relapse, or discontinuation. Where water-scaled metabolite levels were obtained and a Point-RESolved Spectroscopy sequence was utilized, choline, myo-inositol, glutamate + glutamine (Glx), *N*-acetylaspartate, and creatine were measured in the left dorsolateral prefrontal cortex (L-DLPFC) and dorsal anterior cingulate cortex (dACC). An ANCOVA was used to compare metabolites between patients (*n* = 40) and controls (*n* = 46). A linear mixed-model was used to compare olanzapine versus placebo groups.

**Results:**

Cross-sectionally, patients (compared to controls) had higher myo-inositol (standardized mean difference [SMD] = 0.84; 95%CI = 0.25–1.44; *p* = 0.005) in the dACC but not different Glx, choline, *N*-acetylaspartate, and creatine. Longitudinally, patients randomized to placebo (compared to olanzapine) showed a significantly greater change with a reduction of creatine (SMD = 1.51; 95%CI = 0.71–2.31; *p* = 0.0002) in the dACC but not glutamate + glutamine, choline, myo-inositol, and *N*-acetylaspartate.

**Conclusions:**

Patients with remitted psychotic depression have higher myo-inositol than controls. Olanzapine may maintain creatine levels. Future studies are needed to further disentangle the mechanisms of action of olanzapine.

**Supplementary Information:**

The online version contains supplementary material available at 10.1007/s11682-023-00807-0.

## Introduction

Psychotic features, such as delusions and hallucinations, occur in up to 20% of patients with major depressive disorder (MDD) (Coryell et al., 1996; Johnson et al., 1991; Maj et al., 2007; Ohayon & Schatzberg, 2002). Psychotic depression is associated with poorer outcomes including greater disability, increased suicide, and a higher rate of all-cause mortality compared to depression without psychotic features (Coryell et al., 1996; Johnson et al., 1991; Maj et al., 2007; Vythilingam et al., 2003). The neurobiology of psychotic depression is not well understood and there is a paucity of neuroimaging research in this disorder and no published magnetic resonance spectroscopy (MRS) studies to date.

Previous MRS studies indicate that non-psychotic depressed patients have lower levels of choline in the left dorsolateral prefrontal white matter (Wang et al., 2012), of myo-inositol in the dorsolateral prefrontal cortex (DLPFC) and anterior cingulate cortex (ACC) (Chiappelli et al., 2015; Urrila et al., 2017), and lower creatine levels than controls (Moriguchi et al., 2019). An overview of the MRS studies in MDD showed that *N*-acetylaspartate, glutamate, creatine, and phosphocreatine demonstrated a trend of being downregulated (MacDonald et al., 2019); however, the findings were complex and inconsistent. In schizophrenia, there were significant elevations in glutamate + glutamine (Glx) in the basal ganglia and medial temporal lobe but not in the medial prefrontal cortex (mPFC) or DLPFC (Merritt et al., 2016). A meta-analysis of antipsychotic-naïve/free patients with schizophrenia showed lower thalamic *N*-acetylaspartate but no differences in glutamate, choline, myo-inositol, *N*-acetylaspartate, or creatine levels in the DLPFC or medial prefrontal/temporal cortex compared to controls (Iwata et al., 2018).

In relation to the treatment, Chen et al. found that Glx, *N*-acetylaspartate, and myo-inositol levels in the ACC were lower in patients with MDD compared to controls. These metabolite levels were normalized after the treatment with selective serotonin reuptake inhibitors (SSRI) (Chen et al., 2009, 2014). Moreover, Zheng et al. noted that repetitive transcranial magnetic stimulation increased the prefrontal myo-inositol level in treatment-resistant depression. The authors also detected a positive relationship between clinical improvement and myo-inositol increase (Zheng et al., 2010). Similarly, significant increases of myo-inositol and creatine/phosphocreatine in the medial temporal lobe were found in the antipsychotic-treated group, but not in the drug-naïve group, in patients with first-episode schizophrenia spectrum disorder compared to controls (Wood et al., 2008). However, it is not known whether the pathophysiology of psychotic depression resembles or differs from the pathophysiology of non-psychotic MDD or psychosis in the left DLPFC (L-DLPFC) or dorsal ACC (dACC).

Patients with psychotic depression commonly receive treatment with a combination of antidepressant and antipsychotic medication, which complicates the interpretation of MRS studies because antipsychotic medications may affect neurometabolites. Studying participants in the presence and absence of antipsychotics is critical for disambiguating the effects of illness versus antipsychotics on metabolite levels. Moreover, antidepressant mechanisms of action may interact directly or indirectly with antipsychotics. It may also provide new insights into the mechanisms of action of antipsychotic medications in individuals with psychotic depression.

The multi-center Study of the Pharmacotherapy of Psychotic Depression (STOP-PD) II randomized controlled trial (RCT) (Flint et al., 2019) compared sertraline plus olanzapine (*n* = 64) with sertraline plus placebo (*n* = 62) on clinical outcomes in patients with psychotic depression who had attained remission with sertraline plus olanzapine (ClinicalTrials.gov Identifier: NCT01427608). STOP-PD II found that continuing olanzapine was associated with a lower risk of relapse. Moreover, the design of STOP-PD II provided a unique opportunity to investigate the effect of antipsychotics on brain structure, white matter microstructure, and functional connectivity via an integrated magnetic resonance imaging (MRI) study (Bingham et al., 2023; Neufeld et al., 2020, 2023; Voineskos et al., 2020). A subsample of participants in this MRI study completed MRS scans to better understand the pathophysiology of psychotic depression and to understand the mechanism of its treatment with olanzapine. We compared brain metabolite levels at the time of randomization in patients with remitted psychotic depression and non-psychiatric controls in the L-DLPFC and dACC. These regions of interest (ROIs) have been well investigated in previous studies and have particular importance for severe form of depression (Busatto, 2013; Foland-Ross & Gotlib, 2012; Zhang et al., 2017). In the subset of patients who were able to complete their second scan, we conducted an exploratory longitudinal analysis, comparing change in brain metabolites in those randomized to placebo versus those randomized to continue olanzapine in conjunction with depression and delusion symptom severity. We hypothesized that we would find higher glutamate, choline, myo-inositol, *N*-acetylaspartate, and creatine levels in treated patients who achieved remission, based on the previous findings in depression and psychosis. Moreover, we hypothesized that patients randomized to placebo would exhibit a decrease in these metabolite levels compared to those who continued olanzapine.

## Methods

### Participants and study design

Water-scaled MRS data were obtained from the National Institute of Mental Health (NIMH) funded STOP-PD II imaging study (R01 MH099167) conducted at the Centre for Addiction and Mental Health in Toronto (CAMH) and University of Massachusetts (UMass). Water-scaled data was not available from the Nathan Kline Institute nor University of Pittsburgh Medical Centre. The study was approved by each institution’s research ethics board/institutional review board.

Detailed descriptions of the methods of the randomized clinical trial and the integrated neuroimaging study have been published (Flint et al., 2019; Voineskos et al., 2020). Briefly, patients between 18–85 years of age were eligible for STOP-PD II if they met Diagnostics and Statistical Manual of Mental Disorders (Fourth Edition, Text Revision) criteria for current MDD with at least one delusion (with or without hallucinations), had a severely depressed symptom with a total score of ≥ 21 on the 17-item Hamilton Depression Rating Scale (Hamilton, 1960) (HDRS-17), had a score of 3 or higher (i.e., delusion definitely present) on the delusion severity item of the Schedule for Affective Disorders and Schizophrenia (SADS), and did not meet any exclusion criteria (see Supplemental Methods). STOP-PD II comprised three consecutive phases: acute, stabilization, and randomization. First, all participants received open-label acute treatment with sertraline (target dosage, 150–200 mg/day) plus olanzapine (target dosage, 15–20 mg/day) for up to 12 weeks. Second, those who attained remission or near-remission received open-label stabilization treatment with sertraline plus olanzapine for 8 weeks. Third, those who maintained remission or near remission and had a Mini-Mental State Examination score (Folstein et al., 1975) ≥ 24 were eligible for a 36-week double-blind RCT phase that compared the efficacy in preventing relapse of continuing sertraline plus olanzapine (olanzapine group) versus switching to sertraline plus identically appearing placebo pills (placebo group). RCT participants were followed either for 36 weeks or until early termination due to relapse or discontinuation (Supplemental Methods). For patients, neuroimaging data and clinical symptomatology score (i.e., HDRS-17 and SADS) were obtained at two time points: first, at the time of randomization and then at the time of completion or early termination. Healthy controls aged 18–85 years (Supplemental Methods) completed one scan.

### MRS data acquisition and processing

**Fig. 1:**
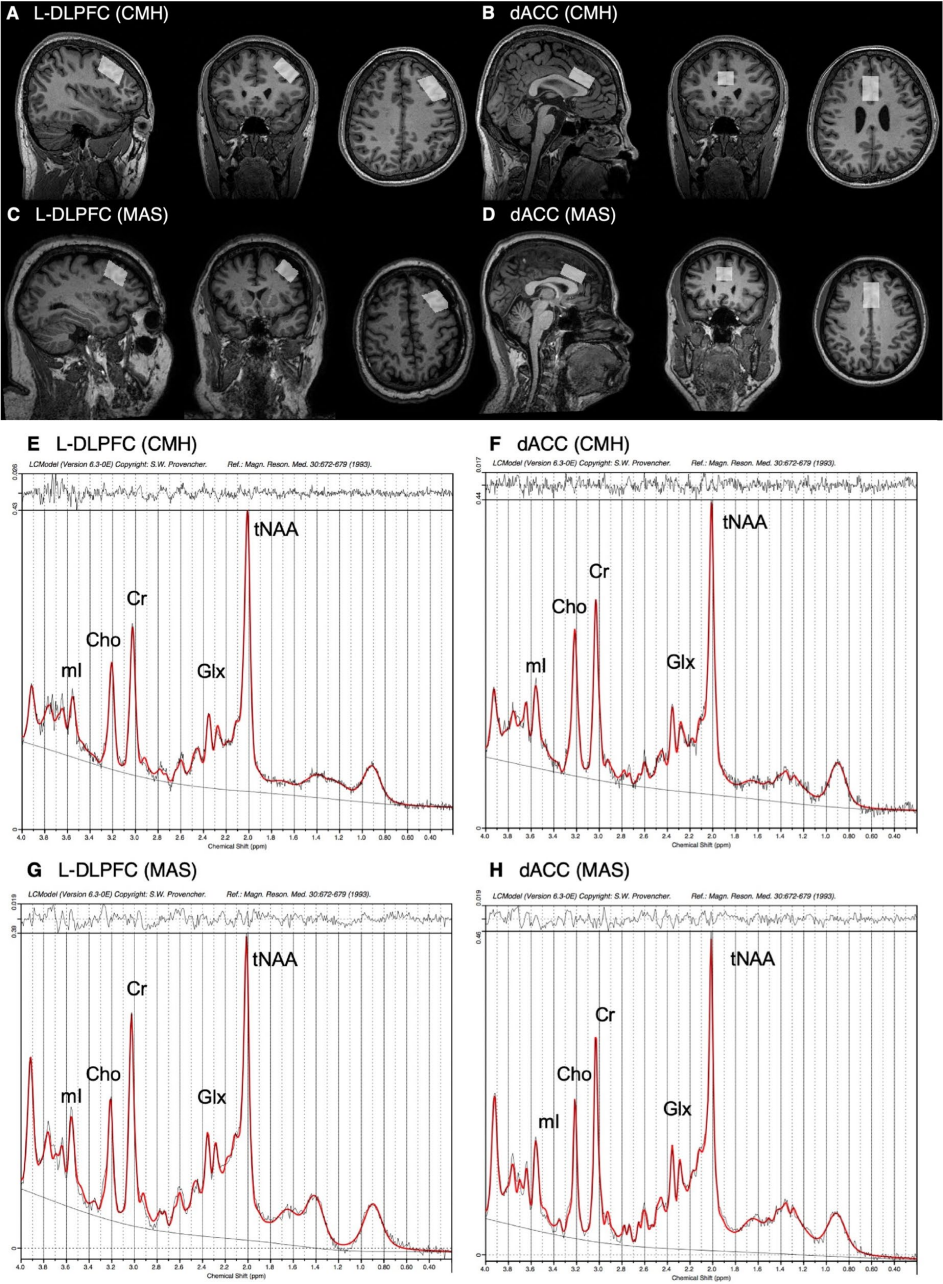
Representative voxel placement and LCModel spectra. **a-d** Representative voxel placement. The L-DLPFC voxel was placed parallel to the line connecting the superior frontal gyrus and the inferior frontal gyrus. The T1-images were acquired sagittally and reformatted to axial and coronal oblique images. The tip of the dACC voxel was placed on top of the anterior part of genu parallel to the cingulate cortex. A sagittal image was acquired parallel to head midline. The voxel dimensions (AP x RL x SI) were as follows. **a** L-DLPFC (CMH): 30 × 15 × 30 mm^3^. **b** dACC (CMH): 30 × 20 × 15 mm^3^. **c** L-DLPFC (MAS): 25 × 25 × 15 mm^3^. **d** dACC (MAS): 30 × 20 × 15 mm^3^. **e–h** Representative LCModel Spectra. **e** L-DLPFC (CMH). **f** dACC (CMH). **g** L-DLPFC (MAS). **h** dACC (MAS). Abbreviations. AP, anterior–posterior; Cho, glycerophosphocholine + phosphocholine; CMH, Centre for Addiction and Mental Health; Cr, creatine + phosphocreatine; dACC, dorsal anterior cingulate cortex; Glx, glutamate + glutamine; L-DLPFC, left-dorsolateral prefrontal cortex; MAS, University of Massachusetts; mI, myo-inositol; ppm, parts per million; RL, right-left; SI, superior-inferior; tNAA, *N*-acetylaspartate + *N*-acetylaspartylglutamate

A Point-RESolved Spectroscopy (PRESS) sequence was utilized for ^1^H-MRS acquisition. Scanner models and acquisition parameters are provided in Table S1. MRS voxels were placed in the L-DLPFC (13.5 mm^3^ [30 mm (AP) x 15 mm (RL) x 30 mm (SI)] for CAMH and 9.375 mm^3^ [25 mm x 25 mm x 15 mm] for UMass) and dACC (9.0 mm^3^ [30 mm x 20 mm x 15 mm] for both sites). The different voxel sizes for the L-DLPFC between sites were chosen due to the scanner configurations. The dACC voxel was placed bilaterally over the midline to obtain sufficient voxel size within a limited scan time. The representative voxel placement at each ROI and site is shown in Fig. 1a-1d. We analyzed water-suppressed spectra with LCModel version 6.3-0E (Provencher, 2001) to obtain the following metabolite levels: Glx, glycerophosphocholine + phosphocholine (Cho), myo-inositol, *N*-acetylaspartate + *N*-acetylaspartylglutamate (total NAA [tNAA]), and creatine + phosphocreatine (Cr). The representative spectra at each ROI and site are shown in Fig. 1e-1h. We checked LCModel spectrum outputs quality. Water-scaled metabolite levels were corrected for voxel tissue composition (i.e., fGM, fWM, fCSF) (Gasparovic et al., 2006). A detailed description of these MRS data acquisition and processing is provided in the Supplemental Methods.

### Statistical analysis

Baseline MRS with water-scaled data were obtained at CAMH and UMass. For the cross-sectional comparison between brain metabolite levels in patients with remitted psychotic depression and controls, we used an analysis of covariance with group (patient versus control) as the independent variable, metabolite levels as the dependent variable, and age, sex, and years of education as covariates. For the longitudinal comparison of MRS measures over time in the olanzapine versus placebo groups, a linear mixed-model regression was used, given that the interval between scans varied among patients. Time (in days) was included, and a treatment-group x time interaction was modeled, with age and sex as covariates. A fixed intercept was included, along with random intercepts to account for within-subject variability. The data were analyzed from CAMH and UMass separately and then harmonized meta-analytically. For multiple comparison corrections, the Bonferroni method was applied. Thus, a significance threshold of p < 0.0025 (= 0.05/20) was applied for metabolite level comparison at each site, given that 5 metabolites × 2 ROIs × 2 sites were examined. Then, we averaged the effect sizes of the dACC data at the two sites by meta-analyzing them as a standardized mean difference (SMD) with a significance threshold of p < 0.01 considering 5 metabolites. The L-DLPFC data were not meta-analyzed due to the different voxel sizes between sites. Pearson correlation coefficients (two-tailed) were evaluated to examine the associations between 1) baseline metabolite levels and treatment outcome/symptom changes, and 2) change in metabolite levels and symptom changes in the whole patient sample, as assessed with the change in HDRS-17 total score for depressive symptoms and the SADS delusion score (Spitzer & Endicott 1979) for psychotic symptoms (Bonferroni correction applied). A detailed description of the statistical analysis is available in the Supplemental Methods.

## Results

### Cross-sectional baseline brain metabolite levels in patients versus controls

The flow diagram of the participants included in this study is presented in Figure S1. The demographic and clinical characteristics of the 40 patients and 46 controls are detailed in Table 1. Patients were older than controls (M = 53.3, SD = 13.9 versus M = 42.2, SD = 16.8; *p* = 0.002). The proportion of males was lower (35.0% versus 56.5%; *p* = 0.046) and the years of education were shorter (M = 13.6, SD = 3.5 versus M = 15.9, SD = 2.8; *p* = 0.001) in patients than in the control group.

**Table 1:** Characteristics of the participants at the baseline scan

*Patients vs. Controls*				
Characteristics	Total (*n* = 86)	Patients (*n* = 40) ^e^	Controls (*n* = 46) ^f^	*p*-value
Age, years^a^	47.4 ± 16.4 (18—78)	53.3 ± 13.9 (22 -78)	42.2 ± 16.8 (18—74)	0.002
Sex, male	40 (46.5%)	14 (35.0%)	26 (56.5%)	0.046
Site, CMH	47 (54.7%)	21 (52.5%)	26 (56.5%)	0.71
Education, years	14.8 ± 3.3 (8—22)	13.6 ± 3.5 (8—22)	15.9 ± 2.8 (11—21)	0.001
HDRS-17 total score	NA	5.2 ± 3.5 (0—13)	NA	NA
CGI-S^a^	NA	1.4 ± 0.7 (1—4)	NA	NA
*Olanzapine vs. Placebo* ^b^				
Characteristics	Total (*n* = 33)	Olanzapine (*n* = 15) ^g^	Placebo (*n* = 18) ^h^	*p*-value
Age, years	54.0 ± 13.6 (22—78)	53.6 ± 13.7 (34 -78)	54.3 ± 14.0 (22—71)	0.89
Sex, male	12 (36.4%)	5 (33.3%)	7 (38.9%)	0.74
Site, CMH	18 (54.5%)	9 (60.0%)	9 (50.0%)	0.57
Education, years ^a^	13.7 ± 3.7 (8—22)	14.1 ± 3.2 (9—20)	13.3 ± 4.1 (8—22)	0.45
Entry status, outpatient	13 (39.4%)	8 (53.3%)	5 (27.8%)	0.13
No. of lifetime suicide attempts ^a^	0.8 ± 1.7 (0—8)	0.6 ± 1.0 (0—3)	0.9 ± 2.2 (0—8)	0.86
Duration of current MDE, months ^a^	11.1 ± 13.6 (0—72)	7.5 ± 5.7 (2—24)	14.2 ± 17.6 (0—72)	0.39
Age of onset	37.4 ± 16.5 (8—68)	38.9 ± 12.7 (10—56)	36.2 ± 19.1 (8—68)	0.66
HDRS-17 total score	4.8 ± 3.5 (0—12)	3.8 ± 2.3 (1—8)	5.6 ± 4.1 (0—12)	0.15
CGI-S ^a^	1.3 ± 0.7 (1—4)	1.1 ± 0.3 (1—2)	1.5 ± 0.9 (1—4)	0.06
SADS delusion score	1 (0)	1 (0)	1 (0)	NA ^i^
HADS anxiety total score	5.0 ± 3.8 (0—14)	5.4 ± 4.0 (1—14)	4.6 ± 3.8 (0—11)	0.56
CIRS total score ^a^	3.1 ± 2.6 (0—9)	2.5 ± 2.6 (0—7)	3.6 ± 2.5 (0—9)	0.22
SAS total score ^a, c^	1.6 ± 2.3 (0—8)	1.3 ± 2.2 (0—6)	1.8 ± 2.4 (0—8)	0.30
BAS global clinical assessment	0 (0)	0 (0)	0 (0)	NA ^i^
AIMS overall severity	0 (0)	0 (0)	0 (0)	NA ^i^
Dose of SER, mg/day ^a^	159.1 ± 29.2 (100—200)	156.7 ± 32.0 (100—200)	161.1 ± 27.4 (100—200)	0.70
Dose of OLZ, mg/day ^a^	14.7 ± 4.1 (5—20)	14.7 ± 4.4 (5—20)	14.7 ± 4.0 (10—20)	0.92
Duration of follow-up, weeks ^a^	24.4 ± 13.1 (2—34)	30.2 ± 7.8 (12—34)	19.3 ± 14.8 (2—34)	0.02
Treatment outcome, relapse ^d^	13 (39.4%)	3 (20.0%)	10 (55.6%)	0.08

Values are shown as N (%) or mean ± SD (range). Notes. a, Mann–Whitney U test was applied; b, for participants with complete longitudinal MRS scans only; c, item7 excluded; d, Fisher’s exact test was applied; e, *n* = 37 (L-DLPFC) and *n* = 39 (dACC); f, *n* = 42 (L-DLPFC) and *n* = 44 (dACC); g, n = 14 (L-DLPFC) and *n* = 15 (dACC); h, *n* = 15 (L-DLPFC) and *n* = 16 (dACC); i, Not available because all the values were the same

Abbreviations. *AIMS*, Abnormal Involuntary Movement Scale; *BAS*, Barnes Akathisia Scale; *CGI-S*, Clinical Global Impression—Severity of Illness; *CIRS*, Cumulative Illness Rating Scale; *CMH*, Centre for Addiction and Mental Health; *dACC*, dorsal anterior cingulate cortex; *HADS*, Hospital Anxiety Depression Scale; *HDRS-17*, 17-item Hamilton Depression Rating Scale; *L-DLPFC*, left dorsolateral prefrontal cortex; *MDE*, major depressive episode; *No*, number; *OLZ*, olanzapine; *SADS*, Schedule for Affective Disorders and Schizophrenia; *SAS*, Simpson Angus Scale; *SER*, sertraline

**Fig. 2:**
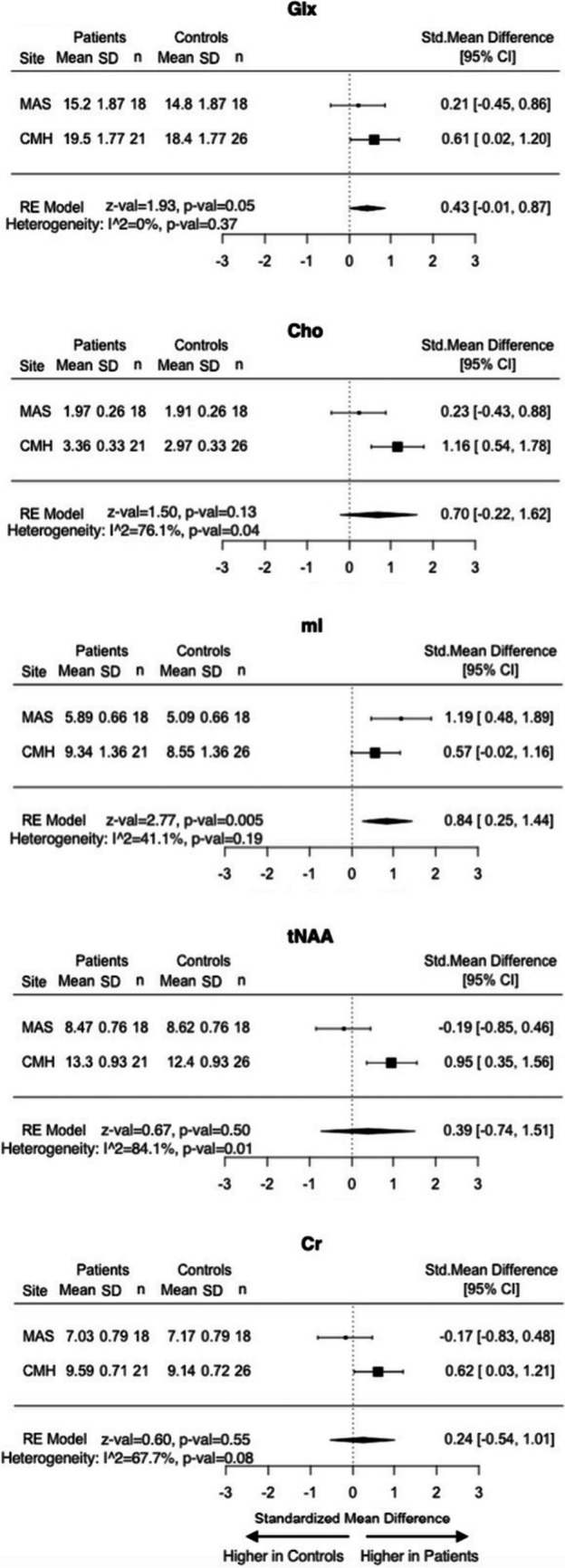
Meta-analysis of metabolite levels in the dACC in patient versus control groups. Mean metabolite levels are estimated marginal means controlled for age and sex. A positive standardized mean difference indicates higher metabolite levels in patients compared to controls. Abbreviations. Cho, glycerophosphocholine + phosphocholine; Cr, creatine + phosphocreatine; dACC, dorsal anterior cingulate cortex; Glx, glutamate + glutamine; mI, myo-inositol; RE, random effect; SD, standard deviation; tNAA, *N*-acetylaspartate + *N*-acetylaspartylglutamate

Site-specific data are presented in Table S3. As shown in Fig. 2, when the data were meta-analyzed, remitted patients with psychotic depression demonstrated higher myo-inositol levels in the dACC (SMD = 0.84; 95% CI = 0.25–1.44; *p* = 0.005; *I*^2^ = 41.1%) than controls after adjusting for age, sex, and years of education. Signal to noise ratio (SNR), full-width at half maximum (FWHM), Cramer-Rao lower bounds (CRLB), and fractions of the three tissue compartments were different between sites in the L-DLPFC due to the different voxel sizes (Table S4a), but they were comparable between patients and controls within sites (Table S4b). When the analyses were limited to adults > 50 years of age, the results were similar (Fig. S2).

### Longitudinal changes in metabolite levels in olanzapine versus placebo groups

Among the 33 patients who completed longitudinal MRS acquisitions, demographic and baseline characteristics were comparable in the olanzapine (*n* = 15) and placebo (*n* = 18) groups (Table 1).

**Fig. 3:**
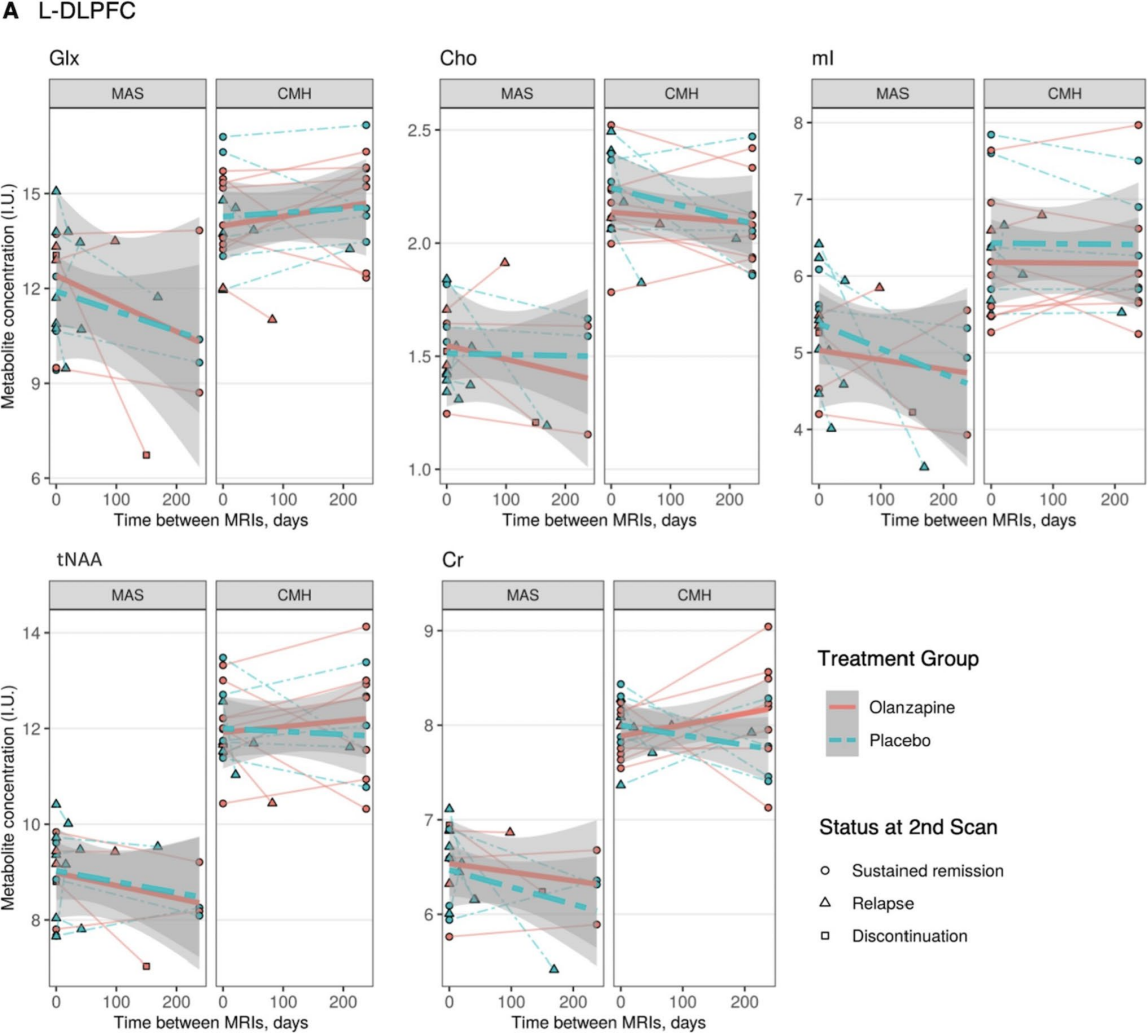

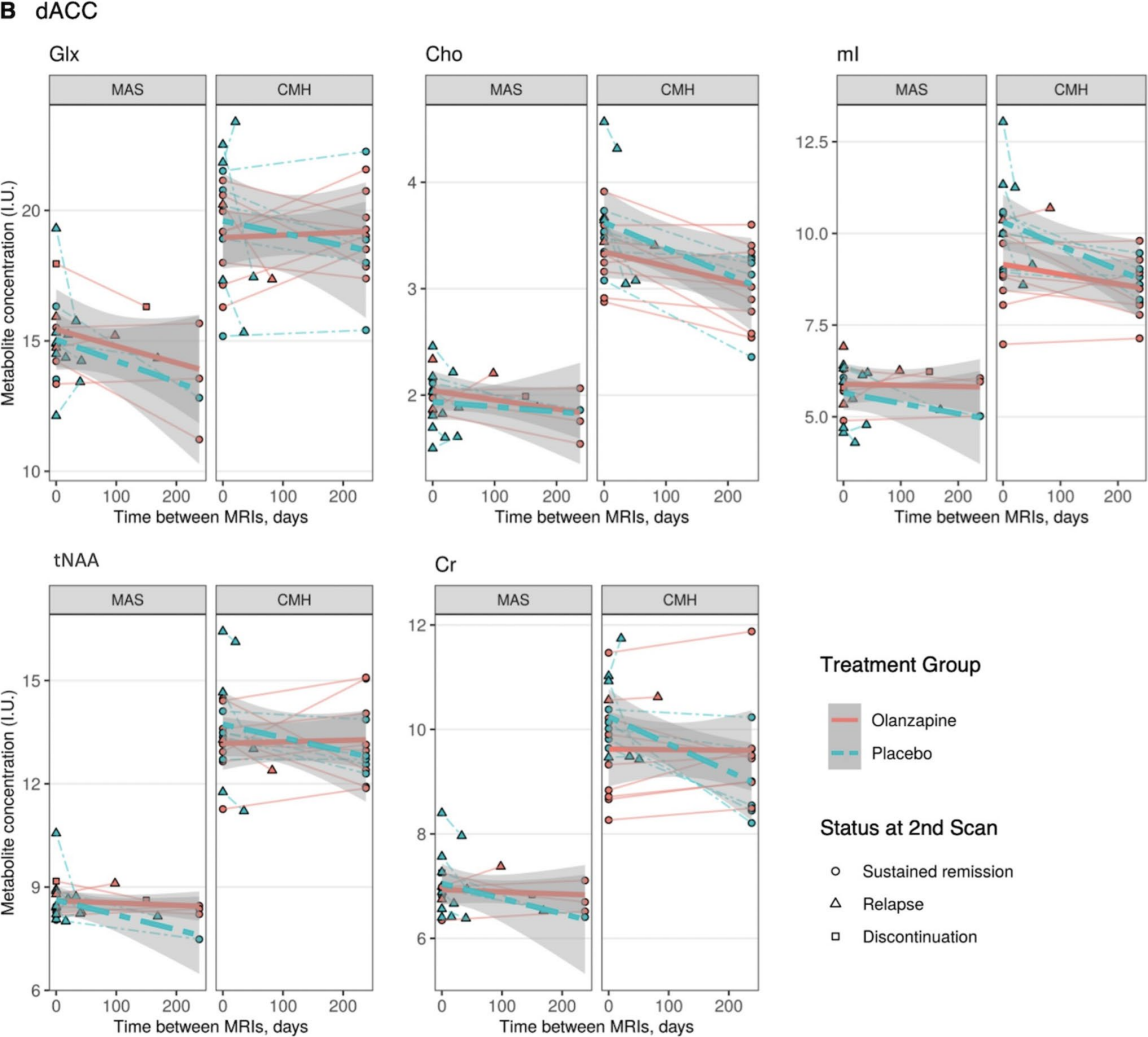
Metabolite level change in olanzapine versus placebo groups, treatment-group x time interaction. **a** L-DLPFC. **b** dACC. Metabolite levels at baseline and at the time of the second scan for each participant (which occurred either at remission, relapse, or discontinuation) are plotted in each panel. In the dACC, the placebo group showed a greater change than the olanzapine group with a decrease in myo-inositol (estimate ± standard error, -0.00465 ± 0.00165; t(12.1) = -2.82; *p* = 0.015) from the UMass data and a decrease in Cr from the CAMH data (-0.00528 ± 0.00148; t(16.4) = -3.56; *p* = 0.003) and UMass data (-0.00329 ± 0.00118; t(11.2) = -2.80; *p* = 0.017). Abbreviations. Cho, glycerophosphocholine + phosphocholine; Cr, creatine + phosphocreatine; dACC, dorsal anterior cingulate cortex; Glx, glutamate + glutamine; I.U., institutional units; L-DLPFC, left-dorsolateral prefrontal cortex; mI, myo-inositol; tNAA, *N*-acetylaspartate + *N*-acetylaspartylglutamate

The absolute changes in metabolite levels at each site are detailed in Table S5 and the treatment-group x time interaction is detailed in Fig. 3 and Table S6. There were no significant differences between groups in the changes of any metabolite levels in the L-DLPFC (Fig. 3a). However, there were significant changes in metabolite levels in the dACC that did not survive correction for multiple comparisons (Fig. 3b). For the CAMH and UMass data, the placebo group showed a greater change with a decrease in Cr than the olanzapine group. Additionally, for the UMass data, the placebo group showed a greater change with a decrease in myo-inositol.

**Fig. 4:**
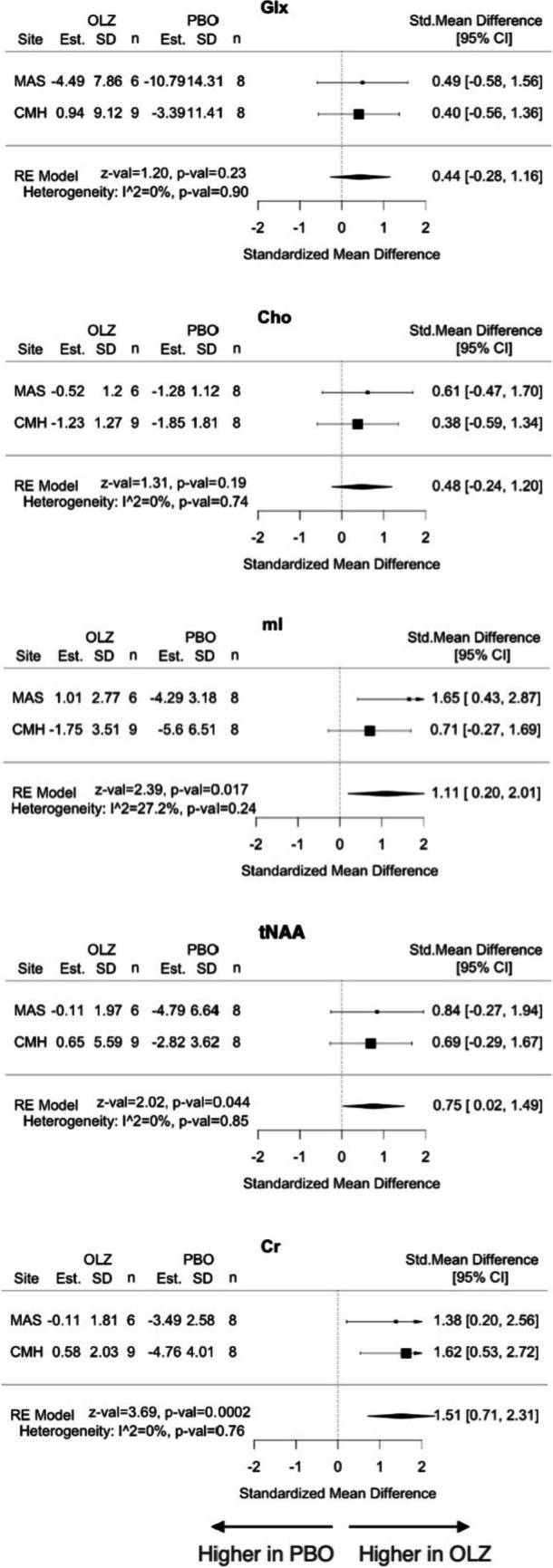
Meta-analysis of metabolite level change in the dACC in olanzapine versus placebo groups. Estimates (Est.) are regression coefficients of time (in days) controlled for age and sex. A positive standardized mean difference indicates a metabolite level increase in the olanzapine group compared to the placebo group (i.e., a decrease in the placebo group compared to the olanzapine group). Abbreviations. Cho, glycerophosphocholine + phosphocholine; Cr, creatine + phosphocreatine; dACC, dorsal anterior cingulate cortex; Est., estimated regression coefficients (× 10^–3^); Glx, glutamate + glutamine; mI, myo-inositol; OLZ, olanzapine; PBO, placebo; RE, random effect; SD, standard deviation (× 10^–3^); tNAA, *N*-acetylaspartate + *N*-acetylaspartylglutamate

When the estimates of the linear mixed-model were meta-analyzed, patients who switched to placebo showed a greater change with a decrease in myo-inositol in the dACC (SMD = 1.11; 95% CI = 0.20–2.01; *p* = 0.017; *I*^2^ = 27.2%), tNAA (SMD = 0.75; 95% CI = 0.02–1.49; *p* = 0.044; *I*^2^ = 0%) and Cr (SMD = 1.51; 95% CI = 0.71–2.31; *p* = 0.0002; *I*^2^ = 0%) levels than those who continued olanzapine (Fig. 4); the change in Cr levels survived after multiple comparisons. SNR, cerebrospinal fluid (CSF) fraction, and CRLB in the L-DLPFC were different between sites but SNR, FWHM, and three tissue compartment fractions in the dACC were comparable between sites (Table S4c). They were comparable between longitudinal scans (Table S4d) or between groups (Table S4e) within sites.

In a sensitivity analysis restricted to patients who sustained remission during the follow-up (*n* = 19), the placebo group showed a decrease in Cr level (SMD = 1.88; 95% CI = 0.55–3.22; p = 0.005; *I*^2^ = 0%) in the dACC compared to the olanzapine group when the data were harmonized across sites, whereas none of the change in other metabolite levels differed significantly between groups (Table S7). In a sensitivity analysis restricted to the placebo group (*n* = 18), patients who relapsed showed a decrease in Cho (SMD = 4.43; 95% CI = 1.83–7.03; p = 0.001; *I*^2^ = 0%), myo-inositol (SMD = 3.78; 95% CI = 1.44–6.11; *p* = 0.002; *I*^2^ = 0%), and tNAA (SMD = 3.37; 95% CI = 1.18–5.55; *p* = 0.003; *I*^2^ = 0%) levels in the dACC compared to those who sustained remission (Table S8). There were no significant correlations between change in any metabolite level and change in the HDRS-17 total score (Table S9) or SADS delusion score (Table S10). Also, there were no associations between any metabolite levels at the time of randomization and the risk of relapse (Table S11), change in the HDRS-17 total score (Table S12) or SADS delusion score (Table S13).

## Discussion

To our knowledge, this is the first MRS study to examine metabolite levels in vivo in patients with psychotic depression. Cross-sectionally, in remitted patients with psychotic depression compared to non-psychiatric controls, we observed higher myo-inositol levels in the dACC. We did not find differences between groups in Glx, Cho, tNAA, and Cr. Longitudinally, patients randomized to placebo demonstrated a decrease in Cr levels in the dACC; the changes in metabolite levels were significantly greater compared to those who remained on olanzapine. We did not find differences between treatment groups in the changes in Glx, Cho, myo-inositol and tNAA. Based on our results, we speculate that olanzapine may sustain remission from psychotic depression by maintaining Cr levels in the dACC.

Taken together, our findings may be explained by the glial cell disruption hypothesis in depression with respect to myo-inositol suppression. Myo-inositol is considered a glial cell marker since it is transported into astrocytes actively (Griffin et al., 2002) and is associated with osmoregulatory functioning in primary astrocytes (Isaacks et al., 1994) as well as neurons (Fisher et al., 2002) that contribute to the maintenance of brain volume. Myo-inositol levels are higher in glia than in neurons (Brand et al., 1993; Urenjak et al., 1993). Several postmortem studies have demonstrated reduced glial density (Cotter et al., 2002; Rajkowska, 2000) in mood disorders, suggesting a histological basis of reduced myo-inositol level in MDD and glial dysfunction. On the other hand, evidence from postmortem studies in patients with schizophrenia suggests an increase in microglia, a marker of neuroinflammation (Trepanier et al., 2016). However, these findings are based on comparisons of cultured neuronal and glial cells, which do not have the same metabolic and active transport phenotypes that are seen in mature neuronal and glial cells.

In human MRS studies in drug-free patients, low myo-inositol level is reported in the ACC in unmedicated patients with MDD (Shirayama et al., 2017; Urrila et al., 2017). Moreover, a recent meta-analysis showed a reduction in mPFC myo-inositol in schizophrenia (Das et al., 2018). However, another meta-analysis that focused on antipsychotic-naïve/free patients found no differences in myo-inositol levels in the DLPFC or mPFC compared to controls (Iwata et al., 2018). This difference may come from the presence of antipsychotic treatment.

With respect to treatment-related metabolite change, previous studies have reported an increase in myo-inositol levels following treatment. Repetitive transcranial magnetic stimulation increased prefrontal myo-inositol in treatment-resistant depression, which was associated with clinical improvement (Zheng et al., 2010). Moreover, Chen et al. reported that reduced Glx, *N*-acetylaspartate, and myo-inositol levels in the ACC were normalized after the treatment with SSRI in patients with MDD (Chen et al., 2009, 2014). Furthermore, treatment with antidepressants increased myo-inositol/creatine level in the L-DLPFC in unmedicated patients with MDD (Kaymak et al., 2009). In relation to the antipsychotics, an 8-week placebo-controlled trial of quetiapine did not find differences in myo-inositol level change in the ACC or L-DLPFC between the medicated and placebo groups in patients with bipolar depression (Chang et al., 2012).

In our study, we found higher myo-inositol levels in the dACC in remitted patients with depression compared to controls. Our study focused on patients who attained remission after treatment with sertraline plus olanzapine. Higher myo-inositol levels may reflect the consequence of this treatment, given that previous studies reported reduced myo-inositol levels in non-psychotic depression and not altered in psychosis, and increases in myo-inositol levels after antidepressant treatments. Future prospective studies are needed to elucidate the effects of antidepressants or placebo on the change in myo-inositol in relation to the treatment outcome, and to identify the role of myo-inositol in depression.

Our longitudinal analysis suggests that olanzapine maintains Cr level in the dACC of patients with remitted psychotic depression. Creatine mainly plays a role in energy metabolism, osmoregulation, and neurotransmission (Rackayova et al., 2017). Our study focuses on olanzapine, which is known to affect lipid metabolism (Spertus et al., 2018). Olanzapine is reported to increase the activity of mitochondrial respiratory chain complexes (Scaini et al., 2013), which works to generate adenosine triphosphate (ATP). Decreased ATP levels have been reported in the frontal lobes in antipsychotic-free patients with schizophrenia (Volz et al., 2000). The lack of phospholipids and impairments in energy metabolism is assumed to be associated with the pathophysiology and the therapeutic target of depression (Kalkman, 2006) and schizophrenia (Albert et al., 2002; Keshavan et al., 2003; Leppik et al., 2020). A recent comprehensive review (MacDonald et al., 2019) and a meta-analysis (Moriguchi et al., 2019) of MRS studies provided evidence for decreased creatine levels in MDD. Reduced phosphocreatine level is also shown in patients with schizophrenia (Volz et al., 2000). In patients with first-episode schizophrenia spectrum disorder, significant increases of creatine and phosphocreatine in the medial temporal lobe were found in the antipsychotic-treated group, but not in the drug-naïve group, compared to controls (Wood et al., 2008). In our sensitivity analysis limited to patients who sustained remission, randomization to placebo was associated with a decrease in Cr compared to randomization to olanzapine; this suggests a direct effect of olanzapine rather than an effect of symptoms on Cr levels. Thus, olanzapine may play a role in maintaining energy metabolism.

Another sensitivity analysis limited to patients who were assigned to placebo found that relapsed patients demonstrated a greater decrease in Cho, myo-inositol, and tNAA in the dACC compared to the sustained remission group. This is consistent with previous studies which reported that successful treatment increased choline and myo-inositol levels (Sonawalla et al., 1999; Zheng et al., 2010, 2015). However, we did not detect a significant correlation between the change in any metabolite levels and symptom severity score changes. Future investigations are required to establish relations between the metabolite level change and clinical outcomes.

Findings from this study should be considered in light of some limitations. First, we were unable to combine the data from all four sites because the excluded two sites did not have water-scaled data and the acquisition parameters such as TE and CRLB of each metabolite were different across sites. Therefore, we harmonized the effect size of the two sites meta-analytically. Since the tissue fractions, FWHM, and SNR were different across the two sites in the L-DLPFC due to different voxel dimensions, and fGM in this voxel was relatively low, our L-DLPFC findings should be interpreted cautiously. Moreover, dACC voxel was placed bilaterally, which would reduce the amount of gray matter and limits our ability to assess the laterality of effects. Second, the relatively small sample size may increase the risk of type II errors. For example, we found a trend level increase in Glx in the dACC in patients than controls (SMD = 0.43 [-0.01–0.87], *p* = 0.05, *I*^2^ = 0%). Third, we did not measure depression score in healthy individuals. They did not have any psychiatric diseases; however, it is possible that healthy controls can have subclinical depressive symptoms. Fourth, some findings were only present at one site. Fifth, there may be potential interaction between sertraline and olanzapine. Sertraline has a weak inhibition of CYP2D6. Active metabolite 2-Hydroxymethyl olanzapine is metabolized via CYP2D6; however, this is assumed to contribute less to olanzapine clearance. Finally, the documented effect of olanzapine on brain metabolite levels in this study may not be directly applicable to other antipsychotics or other illnesses such as bipolar disorder or schizophrenia.

## Conclusion

Myo-inositol levels were higher in the dACC in remitted patients with psychotic depression compared to controls. Continuing olanzapine maintained Cr levels in the dACC. Future placebo-controlled studies with larger sample sizes are needed to confirm the findings from this study, and to examine the relationships between metabolite level changes induced by antipsychotics and their relationship with clinical outcome.

### Supplementary Information

Below is the link to the electronic supplementary material.

Supplementary file1 (PDF 982 KB)

## Data Availability

The datasets generated and/or analyzed during the current study are available from the corresponding author upon reasonable request.
