# Supplementary material for: Brain metabolite levels in remitted psychotic depression with consideration of effects of antipsychotic medication
Source: Brain Imaging Behav. 2023 Nov 2;18(1):117–29. doi: 10.1007/s11682-023-00807-0 (PMC10844359; doi:10.1007/s11682-023-00807-0)
Supplement: Supplementary file 1 — Supplementary file1 (PDF 982 KB) [file 11682_2023_807_MOESM1_ESM.pdf]

Supplemental Methods

Exclusion criteria for patients

Exclusion criteria for patients with psychotic depression included current or lifetime any other psychotic disorder, bipolar disorder, or intellectual disability; current body dysmorphic disorder or obsessive-compulsive disorder, substance abuse/dependence within three months preceding enrollment, and dementia preceding the index episode of depression or a mean score of ≥4 at acute phase baseline on the 26-item Informant Questionnaire on Cognitive Decline in the Elderly (Jorm & Jacomb 1989). Other exclusion criteria included type 1 diabetes mellitus, neurologic disease that might affect neuromuscular function, and unstable physical illness, although many of the study participants had stable chronic physical comorbidities. Participants with standard contraindications for MRI such as metal implants or with acute/unstable physical illnesses were not eligible for the neuroimaging portion of the study.

Exclusion criteria for controls

Healthy (non-psychiatric) controls aged 18–85 years who did not have any psychiatric disorders on the SCID-I/DSM-IV. Participants for healthy controls were excluded if they had any of the following: a neurological disorder (including dementia or head trauma with loss of consciousness), a positive urine toxicology screen, current substance abuse or history of substance dependence within the past six months, or global cognitive impairment, as indicated by a score of <24 on the Mini-Mental State Examination (MMSE) (Folstein et al. 1975). Participants with standard contraindications for MRI such as metal implants or with acute/unstable physical illnesses were not eligible for the neuroimaging portion of the study.

Definition of relapse

Declaring relapse required at least one of the following: 1) enough SCID-rated symptoms to meet criteria for a DSM-IV major depressive episode; 2) HDRS-17 total score of ≥18; 3) SCID-rated psychosis (delusions or hallucinations); or 4) other significant clinical worsening, defined as: i) suicide plan or suicide attempt, ii) development of SCID-rated symptoms of mania or hypomania, or iii) psychiatric hospitalization.

Definition of sustained remission and near remission at the study entry of the RCT phase

Sustained remission at the study entry of the RCT phase was defined as the absence of delusions and hallucinations and a 17-item Ham-D score of ≤10 for two consecutive weeks. Near-remission was defined as the absence of delusions and hallucinations, a Ham-D score of 11-15 with ≥50% reduction in baseline Ham-D score, and being rated as ‘very much improved’ or ‘much improved’ on the Clinical Global Impression (CGI) Scale.

MRS data acquisition

A Point-RESolved Spectroscopy (PRESS) sequence was utilized for 1H-MRS acquisition. Scanner models and acquisition parameters are provided in Table S1. We used data from two of the four sites, CAMH and UMass, because both had unsuppressed water reference files acquired to calculate water-scaled metabolite levels and obtained good quality data. The other two sites did not have unsuppressed water reference files, which precluded our ability to calculate water-scaled metabolite levels. MRS voxels were placed in the left dorsolateral prefrontal cortex (L-DLPFC: 13.5 ml for CAMH and 9.375 ml for UMass) and dorsal anterior cingulate cortex (dACC: 9.0 ml for both sites), regions of particular importance for severe form of depression (Busatto 2013; Foland-Ross & Gotlib 2012; Zhang et al. 2017). The representative voxel placement at each region of interest (ROI) and site is shown in Fig. 1a-1d. All the scans at both sessions followed the acquisition instruction. T1-weighted structural images were acquired to ensure that voxel placements were accurate and correct for partial volume tissue composition. T1-weighted MRI scans were segmented into cerebrospinal fluid (CSF), gray matter (GM), and white matter (WM) using FSL-FMRIB's Automatic Segmentation Tool (FAST) version 5.0 (FMRIB Software Library, Oxford, UK) (Woolrich et al. 2009). Binary voxel masks were created in T1 space by co-registering each participant's MRS voxel to their structural image using Gannet (http://www.gabamrs.com), a MATLAB-based software (The MathWorks, Inc., Natick, MA).

We analyzed water-suppressed spectra with LCModel version 6.3-0E (Provencher 2001). Metabolite levels were calculated using an appropriate basis set by matching the vendor and the TE provided by LCModel to obtain the following metabolite levels: glutamate + glutamine (Glx), glycerophosphocholine + phosphocholine (Cho), myo-inositol, *N*-acetylaspartate + *N*-acetylaspartylglutamate (tNAA), and creatine + phosphocreatine (Cr). The representative spectra at each ROI and site are shown in Fig. 1e-1h.

All the LCModel spectrum outputs were visually checked to verify that baseline did not contain large hills or valleys, that the noise was random and centered on 0, and that the three peaks corresponding to Cho-Cr-NAA were clear and in order. The spectra with a full-width at half maximum (FWHM) $\geq$0.1 ppm, signal to noise ratio (SNR) $\leq$10, or Cramer-Rao lower bounds (CRLB) with %SD values $\geq$15% were considered poor quality and excluded from subsequent analysis. Metabolite concentrations were estimated as institutional units by normalizing each metabolite's peak to the peak of the unsuppressed water signal.

Metabolite concentration correction

Water-scaled metabolite concentrations were corrected for voxel tissue composition. Observed metabolite concentrations (not corrected for metabolite relaxation times) were obtained, relative to a fully relaxed water concentration in tissue [M] by taking the volume fractions, water relaxation times (T1, T2) and water concentrations of the CSF, GM, and WM compartments into account (Gasparovic et al. 2006). We used the following equations:

$$[M] = \frac{[M]_{WS}*[(f_{CSF}*55556*R_{CSF})+(f_{GM}*43300*R_{GM})+(f_{WM}*35880*R_{WM})]}{[0.7*35880*(1-f_{CSF})]}$$

The LCModel performs an operation to give water-scaled data (i.e., $[M]_{WS}$); to reconcile this, (0.7*35880) was added to the denominator of this equation, to undo the assumptions used by the LCModel.

Water relaxation times for each tissue compartment were calculated with the following equation:

$$Ri = (1 - e^{-TR/T1i})*e^{-TE/T2i}, \text{ for } i = CSF, GM, WM.$$

Relaxation times and relative water tissue content values are outlined below.

Compartment	Water concentration (mmol H_2O/kg tissue)	T1 (ms)	T2 (ms)
CSF	55556	4000	2000
GM	43300	1200	100
WM	35880	800	80

Statistical Analysis

Statistical analyses were performed using R (version 3.5.0). To account for potential confounders, we compared the characteristics of (a) the patient and control participants and (b) the participants in the olanzapine and placebo groups, using independent t-tests, chi-square tests, or Fisher's exact test as appropriate. SNR, FWHM, fractions of the three tissue compartments (CSF, GM, WM), and CRLB of each metabolite were compared across sites and within sites between groups. The Shapiro-Wilks test was performed to test the normality of the continuous variable data. When normality assumptions were violated, we applied the Mann-Whitney U test.

For the comparison between brain metabolite levels in patients with remitted psychotic depression and controls, we used an analysis of covariance with group (patient vs. control) as the independent variable, metabolite levels as the dependent variable, and age, sex, and years of education as covariates. For the patients randomized with the assessment of change in MRS measures over time in the olanzapine vs. placebo groups as the main outcome, a linear mixed-model regression was used, given that the interval between scans varied among patients. Time (in days) was included and a treatment group x time interaction was modeled, with age and sex as covariates. A fixed intercept was included, along with random intercepts to account for within-subject variability.

Since different TEs were applied across sites (i.e., 35ms for CAMH and 30ms for UMass), we analyzed data from each site separately and corrected for multiple comparisons using the Bonferroni method. Thus, for metabolite level comparison at each site, a significance threshold of $p < 0.0025$ (=0.05/20) was applied, given that 5 metabolites x 2 ROIs x 2 sites were examined. Then, we averaged the effect sizes of dACC data at the two sites by meta-analyzing them as a standardized mean difference (SMD) with a significance threshold of $p < 0.01$ considering 5 metabolites.

In a sensitivity analysis, we examined whether the results of the metabolite level comparisons between patients and controls were similar in older participants (>50 years of age). We also performed two additional sensitivity analyses: (1) we compared metabolite level changes in the olanzapine vs. placebo groups only in those who had sustained remission at the time of the second scan; and (2) we compared brain metabolite changes in those who relapsed or sustained remission while receiving placebo. Finally, Pearson correlation coefficients were evaluated to examine the associations between 1) baseline metabolite levels and treatment outcome/symptom changes, and 2) change in metabolite levels and change in depressive or psychotic symptoms in the whole patient sample, as assessed with the change in HDRS-17 total score and the Schedule of Affective Disorders and Schizophrenia (SADS) delusion score (Spitzer & Endicott 1979), respectively.

Supplemental References

Busatto, G. F. (2013). Structural and functional neuroimaging studies in major depressive disorder with psychotic features: a critical review. *Schizophr Bull, 39*, 776-786.

Foland-Ross, L. C., & Gotlib, I. H. (2012). Cognitive and neural aspects of information processing in major depressive disorder: an integrative perspective. *Front Psychol, 3*, 489.

Folstein, M. F., Folstein, S. E., & McHugh, P. R. (1975). "Mini-mental state". A practical method for grading the cognitive state of patients for the clinician. *J Psychiatr Res, 12*, 189-198.

Gasparovic, C., Song, T., Devier, D., Bockholt, H. J., Caprihan, A., Mullins, P. G., Posse, S., Jung, R. E., & Morrison, L. A. (2006). Use of tissue water as a concentration reference for proton spectroscopic imaging. *Magn Reson Med, 55*, 1219-1226.

Jorm, A. F., & Jacomb, P. A. (1989). The Informant Questionnaire on Cognitive Decline in the Elderly (IQCODE): socio-demographic correlates, reliability, validity and some norms. *Psychol Med, 19*, 1015-1022.

Provencher, S. W. (2001). Automatic quantitation of localized in vivo 1H spectra with LCModel. *NMR Biomed, 14*, 260-264.

Spitzer, R., & Endicott, J. (1979). Schedule for Affective Disorders and Schizophrenia. 3rd. ed. *New York, NY: New York State Psychiatric Institute, Biometrics Research*.

Woolrich, M. W., Jbabdi, S., Patenaude, B., Chappell, M., Makni, S., Behrens, T., Beckmann, C., Jenkinson, M., & Smith, S. M. (2009). Bayesian analysis of neuroimaging data in FSL. *Neuroimage, 45*, S173-186.

Zhang, B., Li, S., Zhuo, C., Li, M., Safron, A., Genz, A., Qin, W., Yu, C., & Walter, M. (2017). Altered task-specific deactivation in the default mode network depends on valence in patients with major depressive disorder. *J Affect Disord, 207*, 377-383.

Supplemental Tables

Table S1. Magnetic Resonance Imaging acquisition parameters for T1-weighted and proton magnetic resonance spectroscopy scans.

A. Acquisition parameters used for T1-weighted images.

	MAS	CMH
Scanner type	3T Philips Achieva	3T GE Discovery MR750
Head coil	eight-channel	eight-channel
Repetition time (TR), ms	6.7	6.7
Echo time (TE), ms	3.0	3.0
Inversion time, ms	827	650
Flip angle	8°	8°
Field of view, mm	240	230
Matrix	240 x 240	256 x 256
Slice thickness, mm	1.0	0.9
Number of slices	181	200

B. Acquisition parameters used for the proton magnetic resonance spectroscopy (MRS) scan.

	MAS	CMH
Repetition time (TR), ms	2000	2000
Echo time (TE), ms	30	35
Spectra width, Hz	2500	5000
Datapoints	1024	4096
Water-suppressed averages	128	128
Water-unsuppressed averages	128	16
Number of excitations per phase cycle	16	8

Abbreviations. CMH, Centre for Addiction and Mental Health; MAS, University of Massachusetts.

Table S2. Characteristics of the included vs. excluded subjects at the baseline scan.

Patients			
Characteristics	Included (n = 40)	Excluded (n = 30)	p-value
Age, years	53.3 ± 13.9	55.5 ± 17.2	0.55
Sex, male	14 (35.0%)	15 (50.0%)	0.39
Site			<0.001
CMH	21 (52.5%)	6 (20.0%)	
MAS	19 (47.5%)	1 (3.3%)	
NKI	0 (0%)	17 (56.7%)	
PMC	0 (0%)	6 (20.0%)	
Education, years	13.6 ± 3.5	14.8 ± 2.7	0.15
Entry Status, outpatient	16 (40.0%)	7 (24.1%)	0.26
No. of lifetime suicide attempts [a]	0.9 ± 1.6	0.5 ± 1.0	0.46
Duration of current MDE, month [a]	15.0 ± 28.4	10.4 ± 12.0	0.42
Age of onset [a]	36.2 ± 16.3	38.7 ± 21.3	0.90
HDRS-17 total score [a]	5.2 ± 3.5	5.9 ± 3.5	0.33
CGI-S [a]	1.4 ± 0.7	1.2 ± 0.5	0.51
SADS delusion score	1	1	NA [c]
HADS anxiety total score [a]	5.0 ± 3.8	5.2 ± 4.5	0.97
CIRS total score [a]	3.1 ± 2.7	3.8 ± 4.5	0.98
SAS total score [a]	1.4 ± 2.1	1.3 ± 2.7	0.74
BAS global clinical assessment [a]	0	0.03 ± 0.19	0.24
AIMS overall severity [a,b]	0	0.03 ± 0.19	0.24
Dose of SER, mg/day [a]	161.5 ± 29.2	165.5 ± 42.5	0.39
Dose of OLZ, mg/day [a]	15.1 ± 4.1	14.5 ± 5.6	0.88
Duration of follow-up, weeks [a]	21.8 ± 13.9	26.2 ± 11.4	0.25
Treatment outcome, relapse	17 (42.5%)	8 (26.7%)	0.40
Controls			
Characteristics	Included (n = 46)	Excluded (n = 43)	
Age, years [a]	42.2 ± 16.8	45.7 ± 17.1	0.29
Sex, male	27 (57.8%)	21 (48.8%)	0.53
Site			<0.001
CMH	26 (56.5%)	1 (2.3%)	
MAS	20 (43.5%)	0 (0%)	
NKI	0 (0%)	22 (51.2%)	
PMC	0 (0%)	20 (46.5%)	
Education, years [a]	15.9 ± 2.8	15.3 ± 2.5	0.52

Values are shown as N (%) or mean ± SD (range). Notes. a, Mann-Whitney U test was applied; b, item7 excluded; c, Not available because all the values were the same.

Abbreviations. AIMS, Abnormal Involuntary Movement Scale; BAS, Barnes Akathisia Scale; CGI-S, Clinical Global Impression - Severity of Illness; CIRS, Cumulative Illness Rating Scale; CMH, Centre for Addiction and Mental Health; dACC, dorsal anterior cingulate cortex; HADS, Hospital Anxiety Depression Scale; HDRS-17, 17-item Hamilton Depression Rating Scale; L-DLPFC, left dorsolateral prefrontal cortex; MDE, major depressive episode; No, number; OLZ, olanzapine; SADS, Schedule for Affective Disorders and Schizophrenia; SAS, Simpson Angus Scale; SER, sertraline.

Table S3. Metabolite levels in remitted patients vs. controls.

	Patients			Controls			t-value	p-value
	mean [a]	SD	N	mean [a]	SD	N		
L-DLPFC								
Glx								
MAS	12.1	1.47	18	11.9	1.46	20	0.28	0.78
CMH	14.6	1.16	19	15.6	1.16	22	-2.62	**0.01**
Cho								
MAS	1.55	0.23	18	1.43	0.23	20	1.62	0.12
CMH	2.2	0.2	19	1.99	0.2	22	3.19	**0.003**
mI								
MAS	5.33	0.87	18	4.56	0.87	20	2.66	**0.01**
CMH	6.31	0.89	19	5.88	0.9	22	1.48	0.15
tNAA								
MAS	9.07	0.77	18	8.68	0.77	20	1.49	0.14
CMH	12.1	0.9	19	11.9	0.91	22	0.68	0.50
Cr								
MAS	6.55	0.52	18	6.42	0.52	20	0.72	0.47
CMH	7.92	0.46	19	7.97	0.47	22	-0.30	0.77
dACC								
Glx								
MAS	14.8	1.66	18	15.2	1.66	18	-0.77	0.45
CMH	19.5	1.75	21	18.4	1.75	26	2.07	**0.04**
Cho								
MAS	1.95	0.22	18	1.93	0.22	18	0.34	0.74
CMH	3.38	0.33	21	2.96	0.33	26	4.07	**<0.001**
mI								
MAS	5.66	0.63	18	5.33	0.63	18	1.50	0.14
CMH	9.35	1.34	21	8.54	1.35	26	1.96	0.06
tNAA								
MAS	8.42	0.64	18	8.67	0.64	18	-1.13	0.27
CMH	13.4	0.97	21	12.3	0.97	26	3.48	**0.001**
Cr								
MAS	6.9	0.69	18	7.31	0.69	18	-1.71	0.10
CMH	9.61	0.71	21	9.13	0.71	26	2.18	**0.03**

From the CAMH data, patients demonstrated lower Glx (estimated marginal mean±SD, 14.6±1.16 vs. 15.6±1.16, $t_{(3,37)}$=-2.62, p=0.01) and higher Cho (2.20±0.20 vs. 1.99±0.20, $t_{(3,37)}$=3.19, p=0.003) levels in the L-DLPFC, and higher Glx (19.5±1.75 vs. 18.4±1.75, $t_{(3,43)}$=2.07, p=0.04), Cho (3.38±0.33 vs. 2.96±0.33, $t_{(3,43)}$=4.07, p<0.001), tNAA (13.4±0.97 vs. 12.3±0.97, $t_{(3,43)}$=3.48, p=0.001), and Cr (9.61±0.71 vs. 9.13±0.71, $t_{(3,43)}$=2.18, p=0.03) levels in the dACC compared to healthy controls. From the UMass data, patients showed a higher myo-inositol (5.33±0.87 vs. 4.56±0.87, $t_{(3,34)}$=2.66, p=0.01) level in the L-DLPFC but not in the dACC. After the multiple comparison correction, higher Cho and tNAA levels in the dACC from the CAMH data survived statistical differences.

Note. a, estimated marginal means, controlled for age and sex.

Abbreviations. Cho, glycerophosphocholine + phosphocholine; CMH, Centre for Addiction and Mental Health; Cr, creatine + phosphocreatine; dACC, dorsal anterior cingulate cortex; Glx, glutamate + glutamine; L-DLPFC, left dorsolateral prefrontal cortex; MAS, University of Massachusetts; mI, myo-inositol; N, number of subjects; SD, standard deviation; tNAA, *N*-acetylaspartate + *N*-acetylaspartylglutamate.

Table S4. CSF, GM, WM fractions and SNR across sites.

A. Baseline data comparison between sites.

L-DLPFC	CMH (n = 41)		MAS (n = 38)		p-value
	mean	SD	mean	SD	
SNR [a]	32.0	4.7	24.2	2.8	**<0.001**
FWHM [a]	0.06	0.01	0.05	0.01	**<0.001**
fCSF	0.16	0.04	0.23	0.04	**<0.001**
fGM	0.49	0.04	0.44	0.06	**<0.001**
fWM	0.33	0.07	0.30	0.06	0.05
PVC	1.07	0.05	1.19	0.07	**<0.001**
CRLB Glx [a]	0.04	0.01	0.05	0.01	**<0.001**
CRLB Cho [a]	0.02	0	0.04	0.01	**<0.001**
CRLB mI [a]	0.04	0.01	0.05	0.01	**<0.001**
CRLB tNAA [a]	0.02	0	0.03	0	**<0.001**
CRLB Cr [a]	0.02	0	0.03	0	**<0.001**
dACC	CMH (n = 47)		MAS (n = 36)		p-value
	mean	SD	mean	SD	
SNR	21.9	4.6	22.6	2.7	0.44
FWHM [a]	0.04	0.02	0.04	0.01	0.34
fCSF	0.24	0.06	0.22	0.08	0.37
fGM	0.57	0.05	0.57	0.06	0.73
fWM	0.20	0.03	0.21	0.03	0.21
PVC	1.22	0.10	1.26	0.12	0.13
CRLB Glx [a]	0.04	0.01	0.04	0.01	0.20
CRLB Cho [a]	0.02	0.01	0.03	0	**<0.001**
CRLB mI [a]	0.04	0.01	0.05	0.01	**<0.001**
CRLB tNAA [a]	0.02	0	0.03	0	**<0.001**
CRLB Cr [a]	0.02	0	0.03	0	**<0.001**

B. Baseline data comparison between groups within sites.

L-DLPFC	Patients (n = 37)		Controls (n = 42)		p-value
	mean	SD	mean	SD	
MAS					
SNR	23.9	3.12	24.4	2.60	0.59
FWHM [a]	0.05	0.01	0.05	0.01	0.34
fCSF	0.22	0.05	0.23	0.03	0.41
fGM	0.44	0.06	0.45	0.05	0.85
fWM	0.31	0.06	0.28	0.07	0.26
PVC [a]	1.19	0.06	1.19	0.08	0.58
CRLB Glx [a]	0.06	0.01	0.05	0.01	0.16
CRLB Cho [a]	0.04	0.01	0.04	0.01	0.23
CRLB mI [a]	0.04	0.01	0.05	0.01	0.18
CRLB tNAA [a]	0.03	0	0.03	0	0.96
CRLB Cr [a]	0.03	0.01	0.03	0	0.93
CMH					
SNR	31.0	4.84	32.8	4.45	0.22
FWHM [a]	0.06	0.01	0.06	0.01	0.43
fCSF	0.17	0.04	0.16	0.04	0.77
fGM	0.47	0.04	0.51	0.04	**0.002**
fWM	0.35	0.09	0.31	0.06	0.13
PVC	1.07	0.04	1.07	0.05	0.87
CRLB Glx [a]	0.04	0.01	0.04	0.01	0.08
CRLB Cho [a]	0.02	0	0.02	0	0.65
CRLB mI [a]	0.04	0.01	0.04	0.01	0.45
CRLB tNAA [a]	0.02	0	0.02	0	0.54

CRLB Cr [a]	0.02	0	0.02	0	0.16

dACC	Patients (n = 39)		Controls (n = 44)		p-value
	mean	SD	mean	SD	
MAS					
SNR	21.8	3.09	23.3	2.16	0.11
FWHM [a]	0.04	0.01	0.04	0.01	0.20
fCSF	0.23	0.09	0.22	0.07	0.65
fGM	0.56	0.08	0.58	0.05	0.33
fWM	0.21	0.04	0.20	0.03	0.44
PVC	1.27	0.14	1.25	0.1	0.55
CRLB Glx [a]	0.05	0.01	0.04	0.01	0.37
CRLB Cho [a]	0.03	0	0.03	0	0.70
CRLB mI [a]	0.04	0.01	0.05	0	0.16
CRLB tNAA [a]	0.03	0	0.03	0	**0.04**
CRLB Cr [a]	0.03	0	0.03	0.01	0.31
CMH					
SNR	20.6	4.19	22.9	4.66	0.09
FWHM [a]	0.05	0.02	0.04	0.02	0.08
fCSF	0.26	0.05	0.22	0.06	0.07
fGM	0.54	0.04	0.58	0.05	**0.01**
fWM	0.20	0.04	0.19	0.03	0.49
PVC [a]	1.25	0.09	1.21	0.10	0.05
CRLB Glx [a]	0.05	0.01	0.04	0.01	**0.03**
CRLB Cho [a]	0.02	0.01	0.02	0	0.98
CRLB mI [a]	0.04	0.01	0.04	0.01	0.96
CRLB tNAA [a]	0.02	0	0.02	0	0.98
CRLB Cr [a]	0.02	0	0.02	0	0.27

C. Baseline data comparison between sites for patients with complete longitudinal MRS data only.

L-DLPFC	CMH (n = 16)		MAS (n = 13)		p-value
	mean	SD	mean	SD	
SNR	31.0	5.2	23.3	3.3	**<0.001**
FWHM [a]	0.06	0.01	0.05	0.01	0.06
fCSF	0.16	0.04	0.23	0.04	**<0.001**
fGM	0.46	0.04	0.43	0.06	0.09
fWM	0.36	0.08	0.32	0.05	0.09
PVC	1.07	0.04	1.19	0.07	**<0.001**
CRLB Glx [a]	0.04	0.01	0.06	0.01	**0.001**
CRLB Cho [a]	0.02	0	0.04	0.01	**<0.001**
CRLB mI [a]	0.04	0.01	0.05	0.01	**0.005**
CRLB tNAA [a]	0.02	0	0.03	0	**<0.001**
CRLB Cr [a]	0.02	0	0.03	0.01	**<0.001**
dACC	CMH (n = 17)		MAS (n = 14)		p-value
	mean	SD	mean	SD	
SNR	20.9	4.3	21.8	3.2	0.55
FWHM [a]	0.05	0.02	0.04	0.01	0.16
fCSF	0.25	0.06	0.24	0.09	0.66
fGM	0.55	0.03	0.55	0.08	0.98
fWM	0.20	0.04	0.21	0.03	0.33
PVC	1.24	0.10	1.29	0.15	0.34
CRLB Glx [a]	0.05	0.01	0.05	0.01	0.83
CRLB Cho [a]	0.02	0.01	0.03	0	**<0.001**
CRLB mI [a]	0.04	0.01	0.04	0.01	**0.005**
CRLB tNAA [a]	0.02	0	0.03	0	**<0.001**
CRLB Cr [a]	0.02	0	0.03	0	**0.001**

D. Longitudinal scan comparison within sites.

L-DLPFC	pre		post		p-value
	mean	SD	mean	SD	
MAS					
SNR	23.3	3.33	24.5	3.86	0.31
FWHM [a]	0.05	0.01	0.05	0.01	0.08
fCSF	0.32	0.05	0.32	0.06	0.68
fGM	0.43	0.06	0.42	0.05	0.38
fWM	0.23	0.04	0.22	0.03	0.82
PVC	1.19	0.07	1.18	0.05	0.55
CRLB Glx [a]	0.06	0.01	0.06	0.02	0.77
CRLB Cho [a]	0.04	0.01	0.04	0.01	0.62
CRLB mI [a]	0.05	0.01	0.05	0.01	0.52
CRLB tNAA [a]	0.03	0	0.03	0	0.55
CRLB Cr [a]	0.03	0.01	0.03	0	0.15
CMH					
SNR	31.0	5.19	32.7	4.94	0.11
FWHM [a]	0.06	0.01	0.06	0.01	0.48
fCSF	0.36	0.08	0.37	0.10	0.78
fGM	0.46	0.04	0.45	0.05	0.54
fWM	0.16	0.04	0.16	0.04	0.94
PVC	1.07	0.04	1.06	0.04	0.75
CRLB Glx [a]	0.04	0.01	0.04	0.01	0.66
CRLB Cho [a]	0.02	0	0.02	0	0.67
CRLB mI [a]	0.04	0.01	0.04	0.01	0.61
CRLB tNAA [a]	0.02	0	0.02	0	0.32
CRLB Cr [a]	0.02	0	0.02	0	0.33

dACC	pre		post		p-value
	mean	SD	mean	SD	
MAS					
SNR	21.8	3.24	21.4	2.68	0.41
FWHM [a]	0.04	0.01	0.04	0.01	0.29
fCSF	0.21	0.03	0.22	0.03	0.12
fGM	0.55	0.08	0.54	0.07	0.31
fWM	0.24	0.09	0.24	0.08	0.99
PVC	1.29	0.15	1.28	0.12	0.68
CRLB Glx [a]	0.05	0.01	0.05	0.01	0.92
CRLB Cho [a]	0.03	0	0.03	0.01	0.44
CRLB mI [a]	0.04	0.01	0.05	0	0.26
CRLB tNAA [a]	0.03	0	0.03	0	1.00
CRLB Cr [a]	0.03	0	0.03	0	0.29
CMH					
SNR	20.9	4.28	21.7	3.86	0.41
FWHM [a]	0.05	0.02	0.05	0.01	0.08
fCSF	0.20	0.04	0.20	0.04	0.80
fGM	0.55	0.03	0.55	0.04	0.60
fWM	0.25	0.06	0.25	0.07	0.94
PVC	1.24	0.10	1.25	0.12	0.77
CRLB Glx [a]	0.05	0.01	0.04	0.01	0.19
CRLB Cho [a]	0.02	0.01	0.02	0	0.90
CRLB mI [a]	0.04	0.01	0.04	0.01	0.63
CRLB tNAA [a]	0.02	0	0.02	0	0.72
CRLB Cr [a]	0.02	0	0.02	0	1.00

E. Longitudinal scan comparison between groups within sites.

	pre					post					change				
	OLZ		PBO		p-value	OLZ		PBO		p-value	OLZ		PBO		p-value
	mean	SD	mean	SD		mean	SD	mean	SD		mean	SD	mean	SD	
L-DLPFC															
MAS															
SNR	22.2	2.77	24.0	3.63	0.37	22.0	3.87	26.0	3.16	0.07	-0.20	3.9	2.00	3.89	0.34
FWHM [a]	0.05	0.01	0.05	0.01	0.80	0.05	0.01	0.04	0.01	0.46	0	0.01	0.01	0.01	0.74
fCSF	0.24	0.02	0.22	0.05	0.32	0.23	0.03	0.22	0.03	0.50	-0.01	0.03	0	0.07	0.74
fGM	0.42	0.05	0.43	0.06	0.68	0.39	0.03	0.44	0.06	0.19	-0.03	0.04	0	0.03	0.19
fWM	0.3	0.02	0.33	0.06	0.23	0.31	0.07	0.33	0.07	0.59	0.02	0.07	0	0.07	0.74
PVC	1.2	0.06	1.19	0.08	0.95	1.15	0.03	1.2	0.06	0.15	-0.04	0.07	0.01	0.08	0.30
CRLB Glx [a]	0.06	0.01	0.06	0.01	0.97	0.06	0.02	0.06	0.02	0.45	0.06	0.01	0.06	0.01	1.00
CRLB Cho [a]	0.04	0.01	0.04	0.01	0.87	0.04	0.01	0.04	0	1.00	0.04	0.01	0.04	0.01	0.87
CRLB mI [a]	0.05	0.01	0.04	0.01	0.42	0.05	0.01	0.05	0.01	0.88	0.05	0.01	0.04	0.01	0.39
CRLB tNAA	0.03	0.01	0.03	0	0.69	0.03	0	0.03	0	NaN	0.03	0.01	0.03	0	0.69
CRLB Cr [a]	0.03	0.01	0.03	0.01	0.44	0.03	0	0.03	0	NaN	0.03	0.01	0.03	0.01	0.44
CMH															
SNR	33.0	4.24	28.4	5.44	0.08	33.4	4.61	31.7	5.53	0.51	0.44	2.7	3.29	4.96	0.16
FWHM [a]	0.06	0.01	0.07	0.01	0.05	0.06	0.01	0.05	0.01	0.70	0	0.01	-0.01	0.02	0.05
fCSF	0.17	0.04	0.15	0.03	0.36	0.17	0.04	0.14	0.03	0.11	0.01	0.04	-0.01	0.03	0.47
fGM	0.47	0.04	0.44	0.03	0.16	0.46	0.06	0.44	0.03	0.45	-0.01	0.06	0	0.04	0.72
fWM	0.34	0.09	0.39	0.07	0.17	0.33	0.11	0.41	0.05	0.10	0	0.1	0.02	0.08	0.61
PVC	1.07	0.05	1.06	0.03	0.43	1.07	0.05	1.06	0.04	0.58	-0.01	0.07	0	0.04	0.87
CRLB Glx [a]	0.04	0.01	0.05	0.01	0.11	0.04	0.01	0.04	0.01	0.74	0.04	0.01	0.05	0.01	0.14
CRLB Cho [a]	0.02	0	0.02	0	0.41	0.02	0	0.02	0	0.78	0.02	0	0.02	0	0.39
CRLB mI [a]	0.03	0.01	0.04	0.01	0.20	0.04	0.01	0.03	0.01	0.63	0.03	0.01	0.04	0.01	0.21
CRLB tNAA	0.02	0	0.02	0	NaN	0.02	0	0.02	0	0.26	0.02	0	0.02	0	NaN
CRLB Cr [a]	0.02	0	0.02	0	0.27	0.02	0	0.02	0.01	0.64	0.02	0	0.02	0	0.26
dACC															
MAS															
SNR	20.8	3.37	22.5	3.16	0.36	21.2	3.06	21.5	2.56	0.83	0.33	2.07	-1.00	1.6	0.20
FWHM [a]	0.04	0.01	0.04	0.02	0.84	0.04	0.01	0.04	0.01	0.96	0	0.01	0	0.01	0.74
fCSF	0.26	0.11	0.23	0.09	0.58	0.26	0.08	0.23	0.09	0.55	0	0.04	0	0.03	0.96
fGM	0.52	0.07	0.56	0.08	0.36	0.51	0.04	0.56	0.09	0.28	-0.01	0.03	-0.01	0.03	0.73
fWM	0.22	0.05	0.21	0.01	0.51	0.23	0.03	0.21	0.01	0.20	0.01	0.02	0	0.01	0.41
PVC	1.32	0.18	1.26	0.12	0.52	1.3	0.12	1.26	0.13	0.60	-0.02	0.07	0	0.05	0.61
CRLB Glx [a]	0.04	0.01	0.05	0.01	0.77	0.05	0.01	0.04	0.01	0.77	0.04	0.01	0.05	0.01	0.66
CRLB Cho [a]	0.03	0	0.03	0.01	0.43	0.03	0.01	0.04	0.01	0.55	0.03	0	0.03	0.01	0.41
CRLB mI [a]	0.04	0.01	0.04	0.01	0.67	0.04	0.01	0.05	0	0.35	0.04	0.01	0.04	0.01	0.65
CRLB tNAA	0.03	0.01	0.03	0	0.39	0.03	0	0.03	0	0.72	0.03	0.01	0.03	0	0.37
CRLB Cr [a]	0.03	0	0.03	0	0.73	0.03	0	0.03	0	0.25	0.03	0	0.03	0	0.72
CMH															
SNR	21.0	5.27	20.9	3.18	0.95	21.7	3.5	21.6	4.47	0.98	0.67	3.04	0.75	4.06	0.96
FWHM [a]	0.05	0.01	0.06	0.02	0.25	0.04	0.01	0.05	0.02	0.36	0	0.01	-0.01	0.02	0.50
fCSF	0.25	0.05	0.25	0.06	0.94	0.26	0.07	0.24	0.07	0.56	0.01	0.03	-0.01	0.03	0.10
fGM	0.55	0.03	0.54	0.04	0.36	0.55	0.03	0.54	0.05	0.65	0	0.01	0.01	0.02	0.38
fWM	0.19	0.03	0.21	0.04	0.48	0.19	0.04	0.22	0.04	0.16	-0.01	0.02	0.01	0.02	0.16
PVC	1.24	0.08	1.24	0.11	0.95	1.26	0.12	1.23	0.13	0.58	0.02	0.05	-0.02	0.04	0.11
CRLB Glx [a]	0.05	0.01	0.05	0.01	0.68	0.04	0.01	0.04	0.01	0.40	0.05	0.01	0.05	0.01	0.68
CRLB Cho [a]	0.02	0.01	0.02	0.01	0.83	0.02	0.01	0.02	0	0.42	0.02	0.01	0.02	0.01	1.00
CRLB mI [a]	0.04	0.01	0.04	0.01	0.26	0.04	0.01	0.04	0.01	0.42	0.04	0.01	0.04	0.01	0.29
CRLB tNAA	0.02	0	0.02	0.01	0.87	0.02	0	0.02	0.01	0.50	0.02	0	0.02	0.01	0.86
CRLB Cr [a]	0.02	0	0.02	0	0.63	0.02	0	0.02	0.01	0.05	0.02	0	0.02	0	0.61

Note. a, Mann-Whitney U test was applied.

Abbreviations. Cho, glycerophosphocholine + phosphocholine; CMH, Centre for Addiction and Mental Health; Cr, creatine + phosphocreatine; CRLB, Cramer-Rao lower bounds; dACC, dorsal anterior cingulate cortex; fCSF,

fraction of cerebrospinal fluid; fGM, fraction of gray matter; fWM, fraction of white matter; FWHM, full-width at half maximum; Glx, glutamate + glutamine; L-DLPFC, left dorsolateral prefrontal cortex; MAS, University of Massachusetts; mI, myo-inositol; NaN, not a number (all the values are the same); PVC, coefficient of partial volume correction (see Metabolite concentration correction in the Supplemental Methods); SD, standard deviation; SNR, signal noise ratio; tNAA, *N*-acetylaspartate + *N*-acetylaspartylglutamate.

Table S5. Absolute metabolite level changes in olanzapine vs. placebo groups.

	OLZ				PBO					
	pre	post	change	N	pre	post	change	N	t-value	p-value
L-DLPFC										
Glx										
MAS	12.50 ± 1.71	11.21 ± 3.27	-1.29 ± 2.86	5	11.83 ± 1.85	11.82 ± 2.16	-0.01 ± 2.70	8	-0.82	0.43
CMH	14.21 ± 1.27	14.41 ± 1.92	0.19 ± 1.73	9	14.32 ± 1.75	14.44 ± 1.30	0.12 ± 1.08	7	0.16	0.88
Cho										
MAS	1.52 ± 0.18	1.48 ±0.31	-0.04 ± 0.19	5	1.55 ± 0.19	1.46 ± 0.16	-0.09 ± 0.25	8	0.45	0.67
CMH	2.14 ± 0.20	2.09 ± 0.18	-0.05 ± 0.14	9	2.29 ± 0.17	2.08 ± 0.22	-0.22 ± 0.21	7	-0.55	0.59
mI										
MAS	4.97 ± 0.57	5.00 ± 0.86	0.04 ± 0.76	5	5.61 ± 0.65	4.89 ± 0.83	-0.72 ± 0.96	8	1.50	0.16
CMH	6.13 ± 0.80	6.21 ± 0.82	0.07 ± 0.51	9	6.46 ± 0.93	6.39 ± 0.68	-0.07 ± 0.53	7	1.61	0.13
tNAA										
MAS	9.01 ± 0.77	8.61 ± 1.00	-0.40 ± 0.84	5	8.92 ± 1.03	9.14 ± 1.03	0.22 ± 0.79	8	-1.35	0.20
CMH	12.03 ± 0.84	12.07 ± 1.31	0.04 ± 1.15	9	12.16 ± 0.77	11.73 ± 0.85	-0.43 ± 0.98	7	1.28	0.23
Cr										
MAS	6.50 ± 0.48	6.48 ± 0.41	-0.02 ± 0.42	5	6.53 ± 0.46	6.25 ± 0.38	-0.28 ± 0.70	8	0.75	0.47
CMH	7.88 ± 0.25	8.15 ± 0.54	0.27 ± 0.60	9	8.02 ± 0.37	7.79 ± 0.31	-0.23 ± 0.55	7	2.05	0.06
dACC										
Glx										
MAS	15.28 ± 1.60	14.97 ± 2.31	-0.32 ± 1.73	6	15.12 ± 2.10	14.62 ± 1.27	-0.50 ± 2.27	8	0.09	0.93
CMH	19.08 ± 1.63	19.05 ± 1.46	-0.03 ± 2.09	9	19.78 ± 2.50	18.65 ± 2.90	-1.13 ± 2.08	8	1.08	0.30
Cho										
MAS	2.01 ± 0.18	1.97 ± 0.27	-0.04 ± 0.22	6	1.99 ± 0.31	1.89 ± 0.24	-0.10 ± 0.16	8	1.93	0.07
CMH	3.33 ± 0.33	3.06 ± 0.39	-0.27 ± 0.30	9	3.63 ± 0.43	3.22 ± 0.54	-0.41 ± 0.23	8	1.11	0.28
mI										
MAS	5.83 ± 0.71	5.96 ± 0.47	0.13 ± 0.58	6	5.76 ± 0.74	5.44 ± 0.75	-0.33 ± 0.49	8	0.56	0.59
CMH	9.03 ± 1.10	8.70 ± 1.10	-0.33 ± 0.82	9	10.43 ± 1.32	9.14 ± 0.94	-1.29 ± 0.85	8	2.38	**0.03**
tNAA										
MAS	8.54 ± 0.37	8.64 ± 0.38	0.10 ± 0.43	6	8.68 ± 0.83	8.31 ± 0.47	-0.36 ± 0.80	8	0.85	0.41
CMH	13.23 ± 0.96	13.21 ± 1.25	-0.03 ± 1.27	9	13.75 ± 1.39	13.07 ± 1.44	-0.67 ± 0.53	8	1.34	0.20
Cr										
MAS	6.89 ± 0.31	6.91 ± 0.30	0.03 ± 0.31	6	7.11 ± 0.64	6.79 ± 0.53	-0.32 ± 0.32	8	1.70	0.11
CMH	9.55 ± 1.06	9.67 ± 1.01	0.12 ± 0.46	9	10.18 ± 0.57	9.46 ± 1.15	-0.71 ± 0.90	8	2.45	**0.03**

The placebo group showed a decrease in myo-inositol (mean±SD, -0.33±0.82 vs. -1.29±0.85, t(1,15)=2.38, p=0.03) and Cr (0.12±0.46 vs. -0.71±0.90, t(1,15)=2.45, p=0.03) levels in the dACC compared to the olanzapine group from the CAMH data.

Abbreviations. Cho, glycerophosphocholine + phosphocholine; CMH, Centre for Addiction and Mental Health (CAMH); Cr, creatine + phosphocreatine; dACC, dorsal anterior cingulate cortex; Glx, glutamate + glutamine; L-DLPFC, left dorsolateral prefrontal cortex; MAS, University of Massachusetts (UMass); mI, myo-inositol; N, number of subjects; SD, standard deviation; tNAA, *N*-acetylaspartate + *N*-acetylaspartylglutamate.

Table S6. Metabolite level changes in olanzapine vs. placebo groups, treatment-group by time interaction.

Harmonized data	N	SMD	95%CI LB	95%CI UB	I^2	p-value
DLPFC						
Glx	29	-0.04	-0.78	0.70	0%	0.92
Cho	29	0.41	-0.34	1.16	0%	0.28
mI	29	0.69	-0.07	1.46	0%	0.07
tNAA	29	-0.05	-0.80	0.69	0%	0.89
Cr	29	0.61	-0.15	1.37	0%	0.12
dACC						
Glx	31	0.44	-0.28	1.16	0%	0.23
Cho	31	0.48	-0.24	1.20	0%	0.19
mI	31	1.11	0.20	2.01	27.2%	**0.017**
tNAA	31	0.75	0.02	1.49	0%	**0.044**
Cr	31	1.51	0.71	2.31	0%	**0.0002**
Site-specific data	N	Estimate	SE	df	t-value	p-value
DLPFC						
Glx						
MAS	13	-0.00417	0.00754	11.2	-0.553	0.59
CMH	16	0.00044	0.00350	15.2	0.126	0.90
Cho						
MAS	13	0.00033	0.00082	11.0	0.403	0.69
CMH	16	0.00064	0.00046	14.9	1.403	0.18
mI						
MAS	13	0.00483	0.00308	12.5	1.568	0.14
CMH	16	0.00111	0.00121	14.4	0.917	0.37
tNAA						
MAS	13	-0.00219	0.00293	11.0	-0.748	0.47
CMH	16	0.00175	0.00258	15.7	0.679	0.51
Cr						
MAS	13	0.00053	0.00204	13.1	0.257	0.80
CMH	16	0.00237	0.00121	26.0	1.954	0.06
dACC						
Glx						
MAS	14	0.00507	0.00593	12.1	0.855	0.41
CMH	17	0.00454	0.00500	16.9	0.906	0.38
Cho						
MAS	14	0.00062	0.00066	11.8	0.93	0.37
CMH	17	0.00065	0.00075	15.8	0.877	0.39
mI						
MAS	14	0.00465	0.00165	12.1	2.819	**0.015**
CMH	17	0.00387	0.00244	17.0	1.588	0.13
tNAA						
MAS	14	0.00417	0.00234	14.9	1.784	0.09
CMH	17	0.00332	0.00243	16.2	1.364	0.19
Cr						
MAS	14	0.00329	0.00118	11.2	2.797	**0.017**
CMH	17	0.00528	0.00148	16.4	3.56	**0.003**

Main effect of group and time (in days) was estimated in olanzapine group compared to placebo controlling for age and sex. The change in metabolite levels did not differ between olanzapine and placebo groups in the L-DLPFC. In the dACC, the placebo group demonstrated a decrease in myo-inositol from the the UMass data (estimate±standard error (SE), 0.00465±0.00165, t(12.1)=2.82, p=0.015) and Cr from the UMass (0.00329±0.00118, t(11.2)=2.80, p=0.017) and CAMH data (0.00528±0.00148, t(16.4)=3.56, p=0.003). When the data were harmonized across sites,

placebo group showed a decrease in myo-inositol (0.00378±0.00165, $t(32.9)=2.295$, $p=0.028$) and Cr (0.00461±0.00102, $t(29.7)=4.501$, $p<0.001$) compared to olanzapine in the dACC.

Abbreviations. Cho, glycerophosphocholine + phosphocholine; CMH, Centre for Addiction and Mental Health (CAMH); Cr, creatine + phosphocreatine; dACC, dorsal anterior cingulate cortex; df, degree of freedom; Glx, glutamate + glutamine; L-DLPFC, left dorsolateral prefrontal cortex; MAS, University of Massachusetts (UMass); mI, myo-inositol; N, number of subjects; SE, standard error; tNAA, *N*-acetylaspartate + *N*-acetylaspartylglutamate.

Table S7. Metabolite level changes in olanzapine vs. placebo groups for patients who achieved sustained remission during the RCT phase, treatment-group by time interaction.

Harmonized data	N	SMD	95%CI LB	95%CI UB	I^2	p-value
DLPFC						
Glx	17	0.47	-0.55	1.49	0%	0.37
Cho	17	0.47	-0.54	1.48	0%	0.36
mI	17	0.73	-0.29	1.77	0%	0.16
tNAA	17	0.31	-0.69	1.32	0%	0.54
Cr	17	0.80	-0.24	1.84	0%	0.13
dACC						
Glx	18	0.56	-0.58	1.70	0%	0.33
Cho	18	0.24	-0.88	1.37	0%	0.67
mI	18	0.66	-0.48	1.81	0%	0.26
tNAA	18	0.58	-0.56	1.72	0%	0.32
Cr	18	1.88	0.55	3.22	0%	**0.005**
Site-specific data	N	Estimate	SE	df	t-value	p-value
DLPFC						
Glx						
MAS	5	0.00481	0.00282	2.0	1.708	0.23
CMH	12	0.00173	0.00418	10.0	0.414	0.69
Cho						
MAS	5	0.00018	0.00029	2.0	0.618	0.60
CMH	12	0.00031	0.00034	14.4	0.903	0.38
mI						
MAS	5	0.00400	0.00328	2.1	1.221	0.34
CMH	12	0.00145	0.00126	10.0	1.154	0.28
tNAA						
MAS	5	-0.00059	0.00314	3.0	-0.188	0.86
CMH	12	0.00243	0.00292	10.0	0.831	0.43
Cr						
MAS	5	0.00065	0.00210	2.0	0.308	0.79
CMH	12	0.00285	0.00150	18.0	1.903	0.07
dACC						
Glx						
MAS	5	0.00941	0.00878	2.1	1.072	0.39
CMH	13	0.00432	0.00408	11.0	1.059	0.31
Cho						
MAS	5	0.00025	0.00078	2.1	0.326	0.77
CMH	13	0.00032	0.00069	11.0	0.46	0.65
mI						
MAS	5	0.00289	0.00166	2.0	1.741	0.22
CMH	13	0.00243	0.00209	11.0	1.163	0.27
tNAA						
MAS	5	0.00292	0.00112	3.0	2.617	0.08
CMH	13	0.00278	0.00260	11.0	1.069	0.31
Cr						
MAS	5	0.00216	0.00121	2.0	1.78	0.21
CMH	13	0.00470	0.00141	11.0	3.34	**0.007**

Main effect of group and time (in days) was estimated in olanzapine group compared to placebo controlling for age and sex in patients who achieved sustained remission. The harmonized data across sites showed that placebo group had decreased Cr levels in the dACC (0.00409±0.00111, $t_{(15.4)}$=3.68, p=0.002) compared to olanzapine group.

Abbreviations. Cho, glycerophosphocholine + phosphocholine; CMH, Centre for Addiction and Mental Health; Cr, creatine + phosphocreatine; dACC, dorsal anterior cingulate cortex; df, degree of freedom; Glx, glutamate +

glutamine; L-DLPFC, left dorsolateral prefrontal cortex; MAS, University of Massachusetts; mI, myo-inositol; N, number of subjects; SE, standard error; tNAA, *N*-acetylaspartate + *N*-acetylaspartylglutamate.

Table S8. Metabolite level changes in remission vs. relapse for patients who were randomized to the placebo group, treatment-group by time interaction.

Harmonized data	N	SMD	95%CI LB	95%CI UB	I^2	p-value
DLPFC						
Glx	15	-0.29	-1.34	0.76	0%	0.59
Cho	15	1.24	0.11	2.37	0%	**0.03**
mI	15	1.63	-2.34	5.61	87.8%	0.42
tNAA	15	-0.11	-1.14	0.93	0%	0.84
Cr	15	0.28	-2.35	2.92	81.2%	0.83
dACC						
Glx	16	1.55	-0.07	3.18	0%	0.06
Cho	16	4.43	1.83	7.03	0%	**0.001**
mI	16	3.78	1.44	6.11	0%	**0.002**
tNAA	16	3.37	1.18	5.55	0%	**0.003**
Cr	16	0.58	-0.88	2.04	0%	0.43
**Site specific-data**	**N**	**Estimate**	**SE**	**df**	**t-value**	**p-value**
DLPFC						
Glx						
MAS	8	0.00284	0.00751	5.7	0.379	0.72
CMH	7	-0.00496	0.00537	5.4	-0.924	0.39
Cho						
MAS	8	0.00215	0.00100	7.2	2.156	0.07
CMH	7	0.00209	0.00104	5.1	2.019	0.10
mI						
MAS	8	0.01268	0.00221	5.7	5.748	**0.001**
CMH	7	-0.00117	0.00263	5.0	-0.446	0.67
tNAA						
MAS	8	-0.00274	0.00494	6.1	-0.555	0.60
CMH	7	-0.00151	0.00528	8.0	-0.286	0.78
Cr						
MAS	8	0.00798	0.00254	7.4	3.148	**0.015**
CMH	7	-0.00329	0.00242	8.00	-1.359	0.21
dACC						
Glx						
MAS	8	-0.00630	0.01085	6.2	-0.580	0.58
CMH	8	0.06807	0.02605	6.2	2.613	**0.04**
Cho						
MAS	8	0.00057	0.00083	5.6	0.680	0.52
CMH	8	0.01194	0.00339	6.0	3.525	**0.01**
mI						
MAS	8	0.00314	0.00202	5.6	1.554	0.17
CMH	8	0.04527	0.01284	6.2	3.527	**0.01**
tNAA						
MAS	8	0.00252	0.00497	7.8	0.507	0.63
CMH	8	0.02390	0.00663	6.0	3.604	**0.01**
Cr						
MAS	8	0.00295	0.00160	5.3	1.840	0.12
CMH	8	0.01525	0.01245	6.3	1.225	0.26

Main effect of group and time (in days) was estimated in the sustained remission group compared to the relapsed group controlling for age and sex in patients who were assigned to the placebo group. The harmonized data across sites showed that relapsed patients has decreased Cho levels in the L-DLPFC (-0.00188±0.00067, $t_{(14.3)}$=-2.80, p=0.01) compared to sustained remission group.

Abbreviations. Cho, glycerophosphocholine + phosphocholine; CMH, Centre for Addiction and Mental Health; Cr,

creatine + phosphocreatine; dACC, dorsal anterior cingulate cortex; df, degree of freedom; Glx, glutamate + glutamine; L-DLPFC, left dorsolateral prefrontal cortex; MAS, University of Massachusetts; mI, myo-inositol; N, number of subjects; SE, standard error; tNAA, *N*-acetylaspartate + *N*-acetylaspartylglutamate.

Table S9. Correlation between metabolite level change and change in HDRS-17 total score.

Site	N	Estimate	SE	t-value	p-value
L-DLPFC					
Glx					
Whole sample	29	-0.28	0.89	-0.32	0.75
MAS	13	-0.45	1.01	-0.45	0.67
CMH	16	0.72	2.22	0.33	0.75
Cho					
Whole sample	29	3.02	9.60	0.32	0.76
MAS	13	19.99	10.55	1.89	0.09
CMH	16	-40.78	17.85	-2.29	**0.04**
mI					
Whole sample	29	2.17	2.41	0.90	0.38
MAS	13	1.70	3.08	0.55	0.59
CMH	16	6.70	5.69	1.18	0.26
tNAA					
Whole sample	29	-0.77	1.91	-0.40	0.69
MAS	13	1.82	3.30	0.55	0.60
CMH	16	-2.33	2.67	-0.87	0.40
Cr					
Whole sample	29	1.05	3.10	0.34	0.74
MAS	13	1.89	5.25	0.36	0.73
CMH	16	1.71	6.49	0.26	0.80
dACC					
Glx					
Whole sample	31	-0.88	0.76	-1.15	0.26
MAS	14	-0.45	1.38	-0.33	0.75
CMH	17	-1.16	1.11	-1.04	0.32
Cho					
Whole sample	31	6.89	5.91	1.17	0.27
MAS	14	14.60	13.09	1.12	0.29
CMH	17	1.46	8.51	0.17	0.87
mI					
Whole sample	31	0.79	1.90	0.42	0.68
MAS	14	0.58	4.31	0.13	0.90
CMH	17	0.39	2.48	0.16	0.88
tNAA					
Whole sample	31	-1.46	1.73	-0.84	0.41
MAS	14	-3.69	4.02	-0.92	0.38
CMH	17	-0.95	2.34	-0.41	0.69
Cr					
Whole sample	31	1.62	2.42	0.67	0.51
MAS	14	-1.15	7.37	-0.16	0.88
CMH	17	2.53	2.93	0.86	0.40

Positive estimate value indicates positive correlation between metabolite level change and change in HDRS-17 total score controlled for age and sex.

Abbreviations. Cho, glycerophosphocholine + phosphocholine; CMH, Centre for Addiction and Mental Health; Cr, creatine + phosphocreatine; dACC, dorsal anterior cingulate cortex; df, degree of freedom; Glx, glutamate + glutamine; HDRS-17, 17-item Hamilton Depression Rating Scale; L-DLPFC, left dorsolateral prefrontal cortex; MAS, University of Massachusetts; mI, myo-inositol; N, number of subjects; SE, standard error; tNAA, *N*-acetylaspartate + *N*-acetylaspartylglutamate.

Table S10. Correlation between metabolite level change and change in SADS delusion score.

Site	N	Estimate	SE	t-value	p-value
L-DLPFC					
Glx					
Whole sample	29	-0.13	0.16	-0.79	0.44
MAS	13	-0.13	0.23	-0.59	0.57
CMH	16	-0.12	0.36	-0.33	0.75
Cho					
Whole sample	29	-2.10	1.75	-1.20	0.24
MAS	13	-1.58	2.80	-0.56	0.59
CMH	16	-6.11	2.96	-2.06	0.06
mI					
Whole sample	29	-0.75	0.43	-1.74	0.09
MAS	13	-1.21	0.59	-2.05	0.07
CMH	16	-0.26	0.97	-0.27	0.79
tNAA					
Whole sample	29	0.25	0.36	0.69	0.50
MAS	13	0.90	0.71	1.28	0.23
CMH	16	-0.07	0.44	-0.16	0.88
Cr					
Whole sample	29	-0.33	0.58	-0.57	0.57
MAS	13	-1.15	1.15	-1.01	0.34
CMH	16	0.09	1.05	0.08	0.94
dACC					
Glx					
Whole sample	31	-0.24	0.15	-1.59	0.12
MAS	14	-0.06	0.33	-0.19	0.86
CMH	17	-0.40	0.16	-2.46	**0.03**
Cho					
Whole sample	31	0.31	1.16	0.27	0.79
MAS	14	-1.57	3.29	-0.48	0.64
CMH	17	0.35	1.44	0.24	0.81
mI					
Whole sample	31	-0.29	0.37	-0.79	0.44
MAS	14	-1.76	0.87	-2.02	0.07
CMH	17	-0.02	0.42	-0.06	0.96
tNAA					
Whole sample	31	-0.41	0.34	-1.20	0.24
MAS	14	-0.88	0.96	-0.92	0.38
CMH	17	-0.46	0.38	-1.24	0.24
Cr					
Whole sample	31	-0.41	0.48	-0.85	0.40
MAS	14	-1.53	1.70	-0.90	0.39
CMH	17	-0.33	0.50	-0.66	0.52

Positive estimate value indicates positive correlation between metabolite level change and change in SADS delusion score controlled for age and sex.

Abbreviations. Cho, glycerophosphocholine + phosphocholine; CMH, Centre for Addiction and Mental Health; Cr, creatine + phosphocreatine; dACC, dorsal anterior cingulate cortex; df, degree of freedom; Glx, glutamate + glutamine; L-DLPFC, left dorsolateral prefrontal cortex; MAS, University of Massachusetts; mI, myo-inositol; N, number of subjects; SADS, Schedule for Affective Disorders and Schizophrenia; SE, standard error; tNAA, *N*-acetylaspartate + *N*-acetylaspartylglutamate.

Table S11. Association between baseline metabolite level and the treatment outcome for patients who were randomized to the placebo group.

Site	N	Estimate	SE	z-value	p-value
L-DLPFC					
Glx					
Whole sample	15	0.20	0.29	0.69	0.49
MAS	8	-0.57	0.44	-1.30	0.19
CMH	7	1.92	1.06	1.82	0.07
Cho					
Whole sample	15	1.35	1.57	0.86	0.39
MAS	8	2.06	3.48	0.59	0.55
CMH	7	-4.56	4.41	-1.03	0.30
mI					
Whole sample	15	1.22	1.01	1.21	0.23
MAS	8	-1.53	1.46	-1.05	0.29
CMH	7	1.03	1.05	0.98	0.33
tNAA					
Whole sample	15	0.31	0.34	0.91	0.36
MAS	8	-0.34	0.83	-0.41	0.69
CMH	7	0.41	0.90	0.46	0.65
Cr					
Whole sample	15	0.30	0.68	0.44	0.66
MAS	8	-2.61	1.93	-1.35	0.18
CMH	7	6.52	4.15	1.57	0.12
dACC					
Glx					
Whole sample	16	0.05	0.19	0.27	0.79
MAS	8	-0.30	0.43	-0.70	0.48
CMH	8	-0.42	0.50	-0.85	0.39
Cho					
Whole sample	16	0.26	0.68	0.38	0.70
MAS	8	1.18	3.17	0.37	0.71
CMH	8	-3.56	2.81	-1.27	0.21
mI					
Whole sample	16	0.04	0.25	0.15	0.88
MAS	8	-0.46	1.34	-0.34	0.73
CMH	8	-2.43	1.71	-1.42	0.16
tNAA					
Whole sample	16	0.09	0.22	0.39	0.69
MAS	8	-20.25	29.35	-0.69	0.49
CMH	8	-0.26	0.70	-0.38	0.71
Cr					
Whole sample	16	0.16	0.36	0.44	0.66
MAS	8	-1.57	1.60	-0.98	0.33
CMH	8	-1.03	1.35	-0.77	0.44

Positive estimate value indicates positive association between baseline metabolite level and successful treatment (i.e., non-relapse) controlled for age and sex.

Abbreviations. Cho, glycerophosphocholine + phosphocholine; CMH, Centre for Addiction and Mental Health; Cr, creatine + phosphocreatine; dACC, dorsal anterior cingulate cortex; df, degree of freedom; Glx, glutamate + glutamine; L-DLPFC, left dorsolateral prefrontal cortex; MAS, University of Massachusetts; mI, myo-inositol; N, number of subjects; SE, standard error; tNAA, *N*-acetylaspartate + *N*-acetylaspartylglutamate.

Table S12. Correlation between baseline metabolite level and change in HDRS-17 total score.

Site	N	Estimate	SE	t-value	p-value
L-DLPFC					
Glx					
Whole sample	29	-1.45	0.96	1.52	0.14
MAS	13	0.37	1.76	0.21	0.84
CMH	16	-4.44	1.91	-2.33	**0.04**
Cho					
Whole sample	29	-2.19	4.71	-0.46	0.65
MAS	13	-14.95	14.91	-1.00	0.34
CMH	16	21.06	15.15	1.39	0.19
mI					
Whole sample	29	-1.75	2.15	-0.82	0.42
MAS	13	0.73	4.83	0.15	0.88
CMH	16	-2.07	3.58	-0.58	0.57
tNAA					
Whole sample	29	-1.02	1.00	-1.02	0.32
MAS	13	-4.13	3.59	-1.15	0.28
CMH	16	-1.03	3.99	-0.26	0.80
Cr					
Whole sample	29	-1.79	2.24	-0.80	0.43
MAS	13	0.95	6.79	0.14	0.89
CMH	16	-19.64	15.89	-1.24	0.24
dACC					
Glx					
Whole sample	31	1.00	0.77	1.30	0.21
MAS	14	0.99	1.40	0.71	0.50
CMH	17	1.58	1.18	1.33	0.21
Cho					
Whole sample	31	2.08	4.06	0.51	0.64
MAS	14	-13.47	12.21	-1.10	0.30
CMH	17	8.76	5.68	1.54	0.15
mI					
Whole sample	31	1.70	1.50	1.14	0.28
MAS	14	-3.28	5.42	-0.61	0.56
CMH	17	3.29	1.90	1.73	0.11
tNAA					
Whole sample	31	0.22	1.22	0.18	0.89
MAS	14	4.29	4.06	1.06	0.32
CMH	17	0.62	2.32	0.27	0.79
Cr					
Whole sample	31	0.71	1.92	0.37	0.74
MAS	14	2.86	4.94	0.58	0.58
CMH	17	2.54	4.20	0.61	0.56

Positive estimate value indicates positive correlation between baseline metabolite level and change in HDRS-17 total score controlled for age and sex.

Abbreviations. Cho, glycerophosphocholine + phosphocholine; CMH, Centre for Addiction and Mental Health; Cr, creatine + phosphocreatine; dACC, dorsal anterior cingulate cortex; df, degree of freedom; Glx, glutamate + glutamine; HDRS-17, 17-item Hamilton Depression Rating Scale; L-DLPFC, left dorsolateral prefrontal cortex; MAS, University of Massachusetts; mI, myo-inositol; N, number of subjects; SE, standard error; tNAA, *N*-acetylaspartate + *N*-acetylaspartylglutamate.

Table S13. Correlation between baseline metabolite level and change in SADS delusion score.

Site	N	Estimate	SE	t-value	p-value
L-DLPFC					
Glx					
Whole sample	29	-0.12	0.19	-0.62	0.54
MAS	13	0.14	0.40	0.35	0.74
CMH	16	-0.49	0.34	-1.44	0.18
Cho					
Whole sample	29	-0.31	0.88	-0.35	0.73
MAS	13	-0.09	3.59	-0.03	0.98
CMH	16	0.25	2.63	0.10	0.93
mI					
Whole sample	29	-0.06	0.41	-0.15	0.88
MAS	13	0.86	1.07	0.81	0.44
CMH	16	-0.26	0.58	-0.45	0.66
tNAA					
Whole sample	29	-0.11	0.19	-0.60	0.56
MAS	13	-0.33	0.87	-0.38	0.71
CMH	16	-0.50	0.63	-0.79	0.45
Cr					
Whole sample	29	-0.22	0.42	-0.52	0.61
MAS	13	0.03	1.55	0.02	0.98
CMH	16	-4.94	2.31	-2.14	0.05
dACC					
Glx					
Whole sample	31	-0.04	0.11	-0.38	0.71
MAS	14	-0.22	0.34	-0.66	0.53
CMH	17	0.28	0.20	1.40	0.18
Cho					
Whole sample	31	-0.40	0.39	-1.02	0.32
MAS	14	-3.39	2.90	-1.17	0.27
CMH	17	-0.43	1.04	-0.41	0.69
mI					
Whole sample	31	-0.07	0.15	-0.49	0.63
MAS	14	-0.16	1.32	-0.12	0.91
CMH	17	0.13	0.36	0.36	0.73
tNAA					
Whole sample	31	-0.09	0.12	-0.71	0.48
MAS	14	-0.18	1.02	-0.17	0.87
CMH	17	-0.02	0.39	-0.05	0.95
Cr					
Whole sample	31	-0.13	0.21	-0.62	0.54
MAS	14	-0.81	1.17	-0.69	0.50
CMH	17	0.24	0.72	0.34	0.74

Positive estimate value indicates positive correlation between baseline metabolite level and change in SADS delusion score controlled for age and sex.

Abbreviations. Cho, glycerophosphocholine + phosphocholine; CMH, Centre for Addiction and Mental Health; Cr, creatine + phosphocreatine; dACC, dorsal anterior cingulate cortex; df, degree of freedom; Glx, glutamate + glutamine; L-DLPFC, left dorsolateral prefrontal cortex; MAS, University of Massachusetts; mI, myo-inositol; N, number of subjects; SADS, Schedule for Affective Disorders and Schizophrenia; SE, standard error; tNAA, *N*-acetylaspartate + *N*-acetylaspartylglutamate.

Supplemental Figures

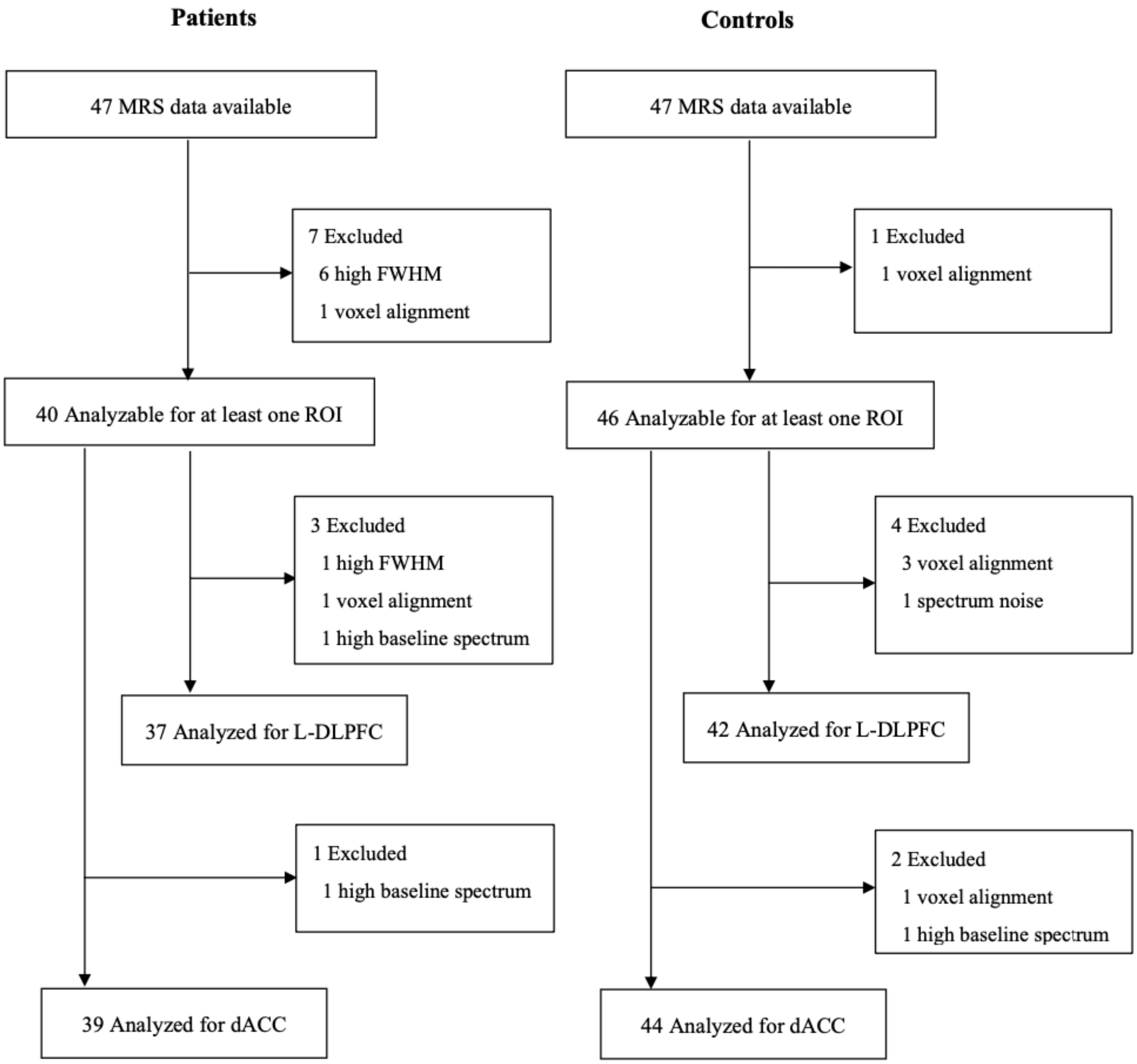

Figure S1a. Flow diagram of the subjects included in this study.

Four sites were involved in the STOP-PD II RCT. At two of the sites (CAMH and UMass), baseline MRS with water-scaled data were obtained that form the basis for the present manuscript. This allowed for 47 patients with water-scaled MRS data, and 47 controls at the same two sites as part of the imaging study. After quality control, seven patients and one control were excluded, allowing for 40 patients and 46 controls with analyzable data for at least one ROI; 37 patients and 42 controls for the L-DLPFC and 39 patients and 44 controls for the dACC. No significant differences in the demographic and clinical characteristics were found in the included vs. excluded participants (Table S2).

Abbreviations. dACC, dorsal anterior cingulate cortex; FWHM, full-width at half maximum; L-DLPFC, left dorsolateral prefrontal cortex; MRS, magnetic resonance spectroscopy; ROI, region of interest;.

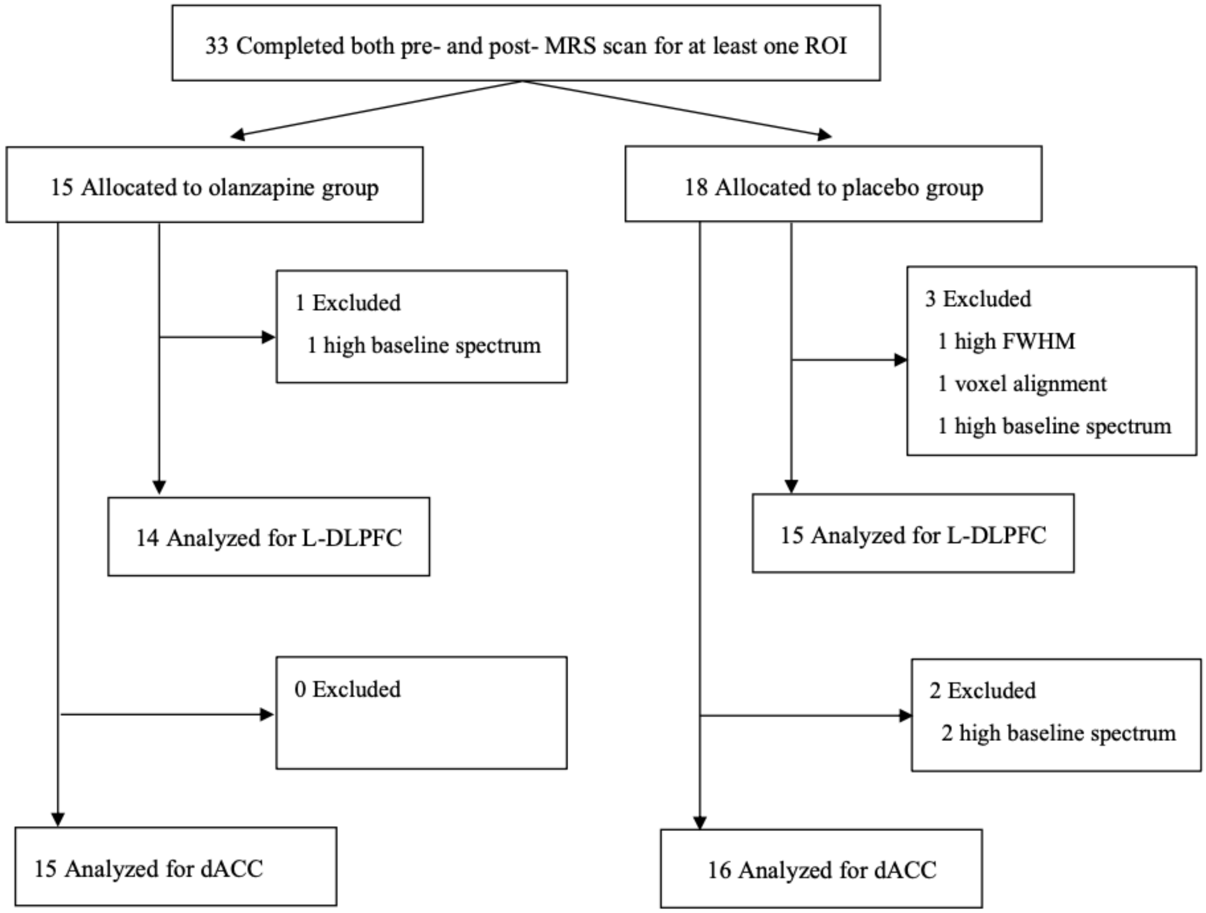

Figure S1b. Flow diagram of the patients included in the RCT.

Among 40 patients included in the analysis of the baseline comparison, 33 patients (15 in the olanzapine group and 18 in the placebo group) completed and acquired qualified data for complete longitudinal scans for at least one ROI; 14 in the olanzapine group and 15 in the placebo group for the L-DLPFC, and 15 in the olanzapine group and 16 in the placebo group for the dACC.

Abbreviations. dACC, dorsal anterior cingulate cortex; FWHM, full-width at half maximum; L-DLPFC, left dorsolateral prefrontal cortex; MRS, magnetic resonance spectroscopy; ROI, region of interest.

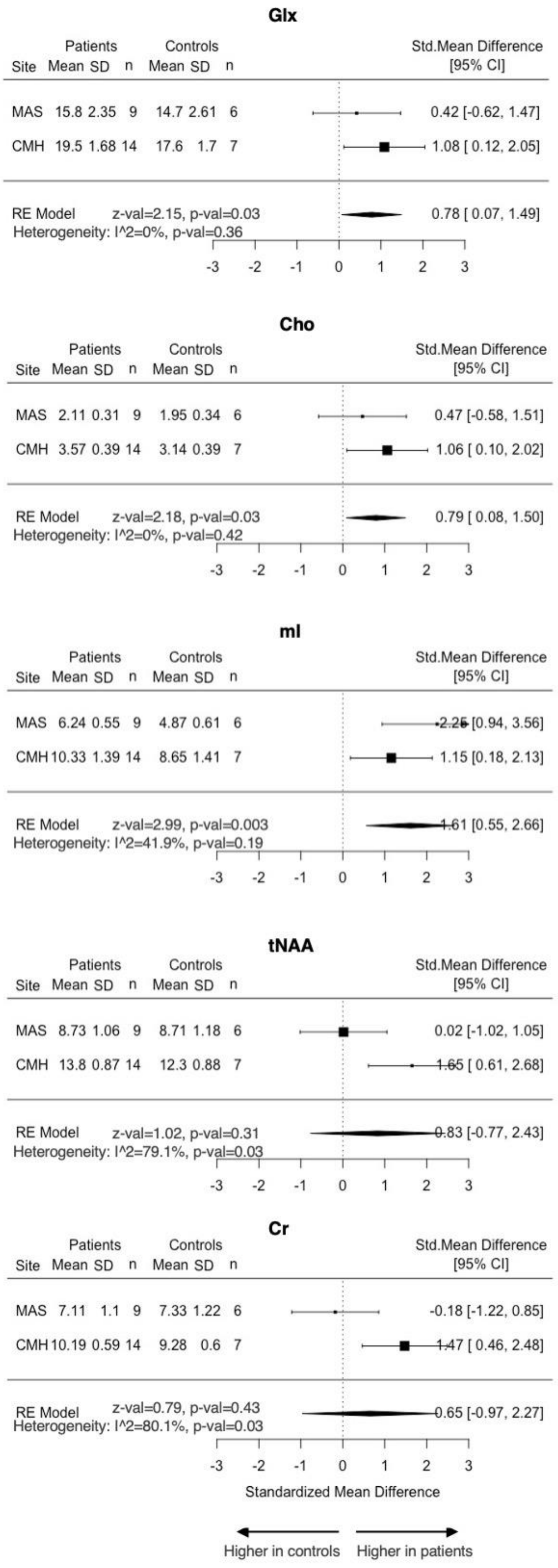

Figure S2. Meta-analysis of metabolite level in the dACC in patient vs. control groups with age of >50 years only.

In people with age of >50 years, patients with remitted psychotic depression demonstrated higher Glx (SMD=0.78; 95% CI=0.07–1.49; p=0.03; I^2=0%), Cho (SMD=0.79; 95% CI=0.08–1.50; p=0.03; I^2=0%) and myo-inositol (SMD=1.61; 95% CI=0.55–2.66; p=0.003; I^2=41.9%) levels in the dACC compared to controls after adjusting for age, sex, and years of education; only myo-inositol level remained significant after multiple comparison correction.

Abbreviations. CMH, Centre for Addiction and Mental Health; Cr, creatine + phosphocreatine; dACC, dorsal anterior cingulate cortex; Glx, glutamate + glutamine; MAS, University of Massachusetts; mI, myo-inositol; n, number of subjects; SD, standard deviation; tNAA, *N*-acetylaspartate + *N*-acetylaspartylglutamate.
